# How to Unmask an Unknown: The Restriction-Modification System *Mho*VII of *Mycoplasma hominis* Expresses Two Complementary Methylation Activities in One Enzyme

**DOI:** 10.3390/ijms27031591

**Published:** 2026-02-05

**Authors:** Lars Vogelgsang, Dana Bäcker, Sebastian Alexander Scharf, Azlan Nisar, Alexander T. Dilthey, Birgit Henrich

**Affiliations:** Institute of Medical Microbiology and Hospital Hygiene, Heinrich-Heine-University Duesseldorf, 40225 Duesseldorf, Germany; lars.vogelgsang@hhu.de (L.V.);

**Keywords:** *Mycoplasma hominis*, RM-system, methylation-sensitive restriction, modified bases, 6mA, nanopore sequencing

## Abstract

Restriction–modification (RM) systems contribute to genome plasticity in *Mycoplasma hominis*, a facultative pathogen with an extremely small but highly heterogeneous genome. The *Mho*VII RM system, which contains a fusion of two methyltransferases (MTases), M1 and M2, was recently identified within a family of Type II RM systems, but its specificity and biological function remained unknown. Phylogenetic analysis revealed that M1 and M2 belong to distinct MTase classes clustering within the *Yhd*J and MTaseD12 branches, respectively. In this study, the dissemination, expression and function of the *Mho*VII system was analyzed in detail using Oxford Nanopore-based methylation analysis, recombinant expression of the individual RM components in *Escherichia coli*, and methylation-sensitive restriction assays. It was thus possible to demonstrate that M1 and M2 methylate the complementary non-palindromic motifs GATG and CATC, and that the associated restriction endonuclease cleaves only DNA lacking 6mA methylation at these sites. The transcriptional analysis of mid-to-late logarithmic cultures indicated a polycistronic organization of the *Mho*VII genes, and GATG/CATC-driven methylation analysis revealed culture-dependent methylation differences, suggesting a post-transcriptional regulation, whereas in the infection of HeLa cells, *Mho*VII transcription was highest at the beginning and was then gradually downregulated in the later stages of infection. These findings establish *Mho*VII as a previously uncharacterized Type II RM system.

## 1. Introduction

*Mycoplasma hominis* is a facultative pathogen belonging to the cell wall-less class mollicutes [1]. In its native environment, the human urogenital tract, it is associated with a range of clinical conditions, including pelvic inflammatory disease and bacterial vaginosis, as well as disseminated infections, such as bacterial arthritis or preterm birth [2,3]. As a result of extensive reductive evolution, *M. hominis* has lost a large proportion of its genome, resulting in the loss of the cell wall and dependence on the L-arginine pathway for energy metabolism [1,4,5]. Consequently, it is among the smallest self-replicating organisms known [6].

Despite its minimised genome, it possesses mobile genetic elements and virulence and defense islands, including a large number of restriction–modification (RM) systems [7,8,9]. RM systems generally consist of a restriction endonuclease (REase) that cleaves unmethylated DNA at a specific motif and an accompanying methyltransferase (MTase) that methylates and protects the host DNA at this site. DNA MTases methylate adenine at the N6 position (6mA-methylation) or cytosine at the N4 or N5 position (4mC- or 5mC-methylation) within the recognition motif, thereby protecting the DNA from self-cleavage (post-segregationally killing) [10,11]. The recognition motif is part of the target recognition domain (TRD) that all MTases comprise. Based on the relative position of the variable TRD along with the other conserved subdomains (AdoMet-binding subdomain and the catalytic subdomain), N6-adenine and N4-cytosine MTases are classified to subgroups α- ε, whereas C5-MTases architecture is more conserved and universal [12,13,14,15,16,17,18].

RM systems are classified into Types I–IV. Type I RM systems consist of an MTase and REase with an additional subunit for specificity, and they cleave DNA several thousand base pairs away from the recognition motif [19]. The recognition motif of type III RM systems, which consist of heteromeric complexes of MTase and REase, is usually short but non-palindromic, and type IV RM systems consist exclusively of a REase that cleaves methylated DNA with low specificity [20,21]. Type II RM systems consist of an MTase and an REase that recognize the same DNA motif. They represent the largest group and are the most widely used in biotechnology [22,23].

Type II REases are further subdivided according to the characteristics of the recognition motif, multimerization and cleavage site [18,19]. Many Type IIP REases recognize palindromic sequence motifs 4–6 bp in length (e.g., the GATC specific *Dpn*I RM system) and often act as monomers composed of a single peptide chain (e.g., *Bst*NI or *Nci*I). Type IIS REases, such as *Fok*I or *Hga*I, recognize a non-palindromic motif and cleave outside the recognition motif (GGATG(N_9/13_) and GACGC(N_5/10_), respectively) [24,25]. A dimerization of these REases is typically required to cleave both strands at their target sites [26,27]. Similarly, two individual MTases are usually required to methylate each strand of the non-palindromic motif, as hemimethylation was shown in many cases to be insufficient to protect the DNA from cleavage [28]. Type IIG restriction enzymes, the third major kind of Type II enzymes, are mostly large fusion proteins of restriction-and-modification enzymes, which methylate only one strand within either a symmetric or asymmetric recognition site and cut 14–21 bp downstream of the recognition site [29].

MTases that lack an associated REases (so called solitary or orphan MTases) are known to influence gene expression and modulate diverse cellular mechanisms (cell cycle control, DNA repair or mismatch repair) and mediate virulence and bacterial biofilm formation [30,31,32,33,34,35,36,37,38]. These solitary MTases often emerged from RM systems after losing REase functionality and shifting to a more regulatory based function [22,39,40]. While RM systems primarily defend against foreign DNA, they can also adopt functions within the host cell similar to solitary MTases and therefore often undergo tight transcriptional control [41,42,43]. The transcriptional regulation of RM systems is highly diverse and ranges from complex systems, which involve fine-tuned regulation by multiple intrinsic promoters and additional regulatory proteins, to a polycistronic organisation of the RM genes, as documented for the *Hga*I-homolog *Mho*VI in *M. hominis* [44,45,46].

In *M. hominis*, several RM-systems have been described, including homologues of *Dpn*II (*Mho*II), *Sau*3AI (*Mho*III), *Hha*I (*Mho*IV), *Eco*47II (*Mho*V) and *Hga*I (*Mho*VI), as well as solitary MTases such as DAM2 or DCM1 [8]. The specificity of the *Mho*VII system (formally named DCM8/DAM3) had remained unclear. Neither the putative restriction endonuclease could be clearly assigned to a known enzyme of other bacteria, nor could the presence of the methyltransferase gene be correlated to a (new) methylation motif in these *M. hominis* strains. Based on the motif order and characteristics, the methyltransferase was assumed to represent a fusion protein of an N-terminal β group N6-N4-MTase (M1, formerly DCM8) and a C-terminal α group N-6-adenine MTase (M2, formerly DAM3) [8]. A TA-repeat region was detected within the target recognition domain (TRD) of the M2-MTase part, suggesting a phase-variable expression of the full length *Mho*VII MTase (M12-MTase) or the M1-part with an M2-remnant [8].

In this study, the *Mho*VII RM system was characterized in detail, including phylogenetic, transcriptional and functional analysis of each enzyme, leading to the first experimental proof of an RM system acting on the GATG/CATC motif.

## 2. Results

### 2.1. The Composition of the MhoVII RM System Was Highly Conserved in M. hominis

**Figure 1:**
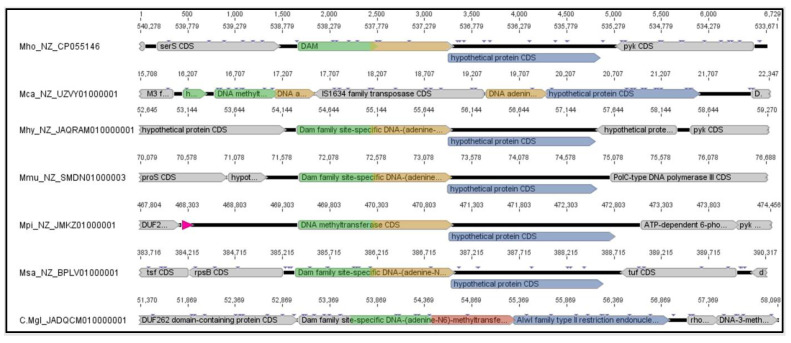
**Scheme of the genomic loci of *mho*VII in Mollicutes.** The genomic region of the *mho*VII-homologue RM genes is schematically shown for *M. hominis* isolate SS10 (Mho), *M. caviae* (Mca), *M. hyosynoviae* (Mhy), *M. mucosicanis* (Mmu), *M. pirum* (Mpi), *M. salivarium* (Msa) and Candidatus *M. glomeromycotorum* (Mgl); abbreviations are given with the respective accession numbers. *Mho*VII MTase genes are coloured in green (M1 region) and orange-red (M2 region), restriction endonuclease genes in blue.

Using Taq-PCR, 64/238 *M. hominis* isolates from our strain collection tested positive for the presence of both MTase parts, M1 and M2, and the restriction endonuclease R.*Mho*VII, corresponding to a prevalence of 26.9%. Blast v2.17.0 analysis revealed the presence of *Mho*VII-homolog systems in further mollicutes, but with integration into other chromosomal genome regions (Figure 1).

The order of MTase and restriction endonuclease genes corresponded to *Mho*VII. Like the MTase M12 of *Mho*VII, most MTases were predicted to be expressed as fusion proteins of two independent 6mA MTases. In *M. caviae*, the MTase gene was fragmented, not least by an integrated IS element. As shown in Table 1, the peptide region of all M1-homologs was classified by NCBI as *Yhd*J ((1.1) CDD:440623) and the M2-homologs as DAM ((2.2) CDD:440107).

**Figure 2:**
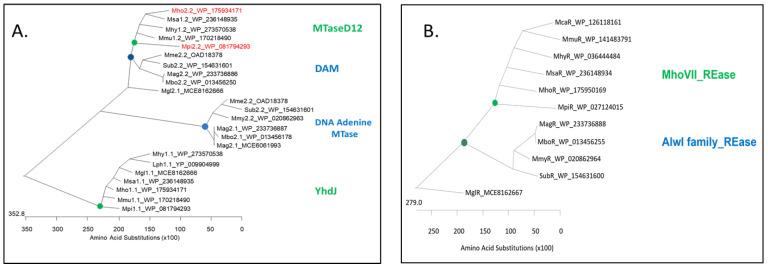
**Phylogenetic branches of *Mho*VII homolog MTase** (**A**) **and REase** (**B**) **enzymes.** The phylogenetic trees were constructed with MegAlign 5.08 of DNAStar; labelling consists of abbreviation of the organism, numbering of MTase region and accession numbers of the protein (Table 1). Branches of *Mho*VII MTases and REase regions are coloured in green, branches of *Alw*I family elements in blue.

Beside *M. hominis*, this constellation was described in *M. pirum* and, due to the fragmentation of the MTase gene, suspected in *M. caviae*. However, we considered the original assignment of the M2 MTase parts to DAM to be incorrect and would assign them to MethyltransfD12, as the respective protein sequences of both *M. hominis* and *M. pirum* clustered in the phylogenetic tree with MTaseD12 members of *M. salivarium*, *M. hyosynoviae* and *M. mucosicanis* (Figure 2A), whose REases also clustered in one branch, therefore named *Mho*VII family-REase (Figure 2B).

*Yhd*J was always fused with a C-terminal Methyltransferase D12 ((1.2) CDD:451538) in mycoplasmas, but occurred alone in the *Lactococcus* phage AM1, which may indicate its origin and route of transmission. MTase fusion with an N-terminal DNA adenine methylase ((2.1) CDD:442619) and a C-terminal DAM ((2.2) CDD:440107) was found in *M. meleagridis*, *M. bovis* and the non-mollicutes *Streptococcus uberis* and, as two separate proteins, in *M. agalactiae*, suggesting a closely related but separate RM system. This was supported by the phylogenetic tree of the REases (Figure 2B): Members of the *Mho*VII RM system always carried the (so called *Mho*VII) REase that clustered in a different branch than the *Alw*I family REases, which were always associated with the fused DNA adenine—Dam MTase.

As the target recognition domain (TRD) of an MTase is responsible for the enzyme’s specific recognition of its target DNA, we compared these regions of both MTase parts of both putative RM systems in a multiple sequence alignment. The phylogenetic separation of *Yhd*J (1.1) and the DNA adenine methylase (2.1) from DAM (2.2) and Methyltransferase D12 (1.2) confirmed the former observation (Supplementary Figure S1A). Although members of (1.2) and (2.2) did not separate clearly in two branches, an additional region of 15 AA was only found in the 5′-TRD region of (2.2)-members and occurred in neither (1.2)-members nor in M2.*Mho*VII (Supplementary Figure S1B), underlining the affiliation of M2.*Mho*VII to the (1.2)-family.

### 2.2. RM.MhoVII Is Polycistronically Organized

**Figure 3:**
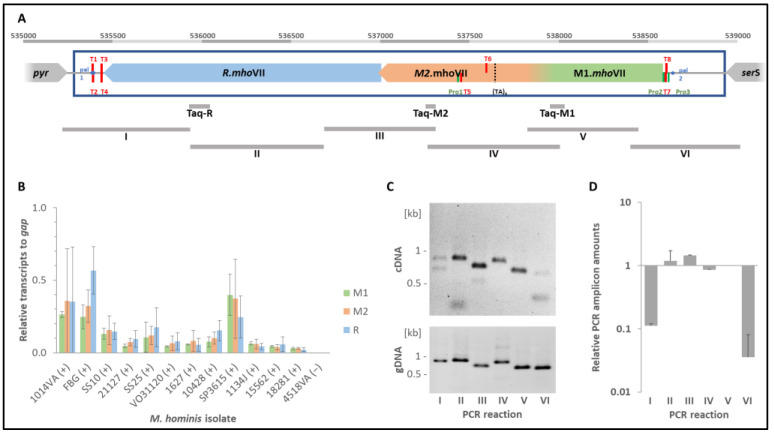
**Transcription of *mho*VII genes in *M. hominis.*** (**A**) Scheme of the *mho*VII cassette (blue box) located between the 3′-ends of the chromosomal *pyr* and *ser* genes, shown for *M. hominis* isolate SS10 (acc. no. CP055146.1). PCR products (grey) are named according to Table 2. Bioinformatically predicted promoters (Pro1-3) are shown in green, terminators (T1-8) in red and palindromic regions as blue dots (pal1-2). (TA)_x_ denotes the variable TA-repeat region in M2.*mho*VII. (**B**) mRNA levels of the *mho*VII genes from 12 *mho*VII positive (+) and one *mho*VII negative isolate (−) were quantified by Taqman-RT-PCR relative to mycoplasmal *gap* using the ΔCT method. Standard deviations were calculated from duplicates of two biological and one technical replicate. (**C**) PCR products I-VI, derived from cDNA (top) and genomic DNA (gDNA; bottom) of isolate SS10, were separated on 1% agarose gels. (**D**) Bar graphs represent the mean and standard deviations of relative transcript levels of I to VI from 12 *mho*VII positive *M. hominis* isolates, which were analyzed in biological replicates, measured in duplicates and normalized to V.

In *M. hominis*, the *Mho*VII gene cassette was always located between *pyr* and *ser*S. Blast analysis of the *Mho*VII genes flanking region revealed that 223/225 nucleotides upstream of the MTase gene belonged to the untranslated 5′-region of the cassette; 136/192 nucleotides between 3′-ends of *pyr* and the REase gene belonged to the cassette (Figure 3A, blue frame), associated with a deletion of 96 nt chromosomal region, corresponding to nt 519267-519362 in PG21 (ATCC23114; NC_013511.1). Bioinformatics analyses were performed using the ProPr 2.0 web tool to identify potential promoter and terminator sites within the gene cassette (see Supplementary Table S2). As shown in Figure 3A, promoter and terminator sites were predicted upstream of the MTase gene and within the M2-part downstream of the (TA) repeat region; additional terminators were predicted downstream of the REase gene.

**Table 2:** PCR primers used.

PCR	Primer Name	Sequence (5′-3′)	Start ^1^	End ^1^
*gap*	Taq_gap_F	GCAGGCTCAATATTTGACTCACT	671913	671935
	Taq_gap_R	GATGATTCATTGTCGTATCATGC	671985	672004
Taq-M1	Taq_M1-F2	ACCGGTAGAGTTAATGGAAAAA	538063	538084
	Taq_M1-R2	TTAAGGCCGCAACRCAAGTC	537981	538000
Taq-M2	Taq_M2-F1	TGAAAATGCAAGAAACAAGAGAA	537304	537326
	Taq_M2-R	GCACCCGAAATTAAGTATGGA	537260	537280
Taq-R	Taq_R-F	CATTGCCAATTTTTAAGGTGGATAAT	536022	536047
	Taq_R-R	TGTTTTAGGGCAATGTATTTTTCTGAT	535900	535926
I	M12_F1	AGGCGAATATGGYGCTAAAAA	535170	535150
	M12_R1	ACCATCAGAAAAATACATTGCCC	535907	535885
II	M12_F2	GGCAATGTATTTTTCTGATGGTGC	535909	535886
	M12_R2	GACACAGACAGCCCGGTT	536668	536651
III	M12_F3	CCGGGCTGTCTGTGTCAA	536670	536653
	M12_R3	TGCAAGAAACAAGAGAAGACGA	537298	537277
IV	M12_F4I	TGCACACTTCTTTTTGCT	535653	535636
	M12_R4I	TGAATTTGCTATTTGAGCAG	536147	536128
V	M12_F4II	GGTTCAGCAACTCTAAATTC	534904	534885
	M12_R4II	TCCCGTAACGAAAAGAGT	535536	535519
VI	M12_F5	TCGCCCAAATAATACAATGGTGA	537856	537834
	M12_R5	TGGCAGATCAGGAATAGACTTTG	538439	538417
VII	M12_F6	CCCAAAGTCTATTCCTGATCTGC	538437	538415
	M12_R6	TCCAAGGTCGTAGGGCAA	538998	538981

^1^ Position in *M. hominis* Ss10 (acc.-no. CP055146.1).

To analyze the transcriptional activity of *Mho*VII, Taq-primers were first used in RT-qPCR for both MTase gene parts (Taq-M1 and Taq-M2) and for the restriction endonuclease gene (Taq-R) (Figure 3A). All RT-PCRs were positive in every *mho*VII positive *M. hominis* isolate but were below the level of *gap* transcripts (Figure 3B). Transcript levels varied between isolates in some cases, but statistically significant differences in transcript abundance were not observed between RM genes within a single isolate. As expected, no transcripts could be detected in the *mho*VII negative control.

To investigate the hypothesis of a polycistronic organization of *Mho*VII genes next, primers were designed to amplify overlapping regions spanning the entire cassette (Figure 3A). As a control of primer pair performance, PCR was first conducted with genomic DNA (gDNA) and cDNA of *M. hominis* isolate SS10 (Figure 3C). Amplicon II–V was detected in similar quantities when using cDNA and gDNA, whereas the amplification of transcription regions I and VI was strongly inhibited only in cDNA. (Figure 3C). The 12 *mho*VII-positive isolates tested showed comparable amplification of the *mho*VII regions II–V, and here, too, the amounts of cDNA amplicons I and VI were significantly lower. The average amounts of amplicons I-V for the 12 *Mho*VII positive isolates are shown in Figure 3D (qPCR data are listed in Table S3). These results are in good accordance with the position of the in silico predicted promoter (Pro2/Pro3) and terminator (T2/T4) regions, whereas the position of the internal promoter (Pro1) or terminators within the cassette was not supported by experimental data. These data clearly demonstrate that *Mho*VII is expressed by a polycistronic mRNA.

### 2.3. M12.MhoVII Methylates the Palindromic Sequence Motif G^m^ATG/C^m^ATC

As genetic engineering of *M. hominis* to create M1- or M2-deficient strains remains technically challenging, we started with the idea of expressing the MTase of *Mho*VII in both parts (rM1 or rM2) or as a fusion protein (rM12) in *E. coli*. Analysis of the change in the DNA methylation pattern of the respective *E. coli* clones with respect to the plasmid-free bacterium should help to identify the methylation motifs of both MTase parts. Blast analysis revealed that *E. coli* DH5aF’IQ itself did not possess these MTases. The respective gene regions were amplified in *M. hominis* isolate SS10 in overlapping fragments that enabled the mutation of TGA to TGG codons and the addition of flanking *Bam*HI-*Hin*DIII sites used for cloning (see Supplementary Table S1). Plasmids were propagated into *E. coli* DH5αF’IQ (New England Biolabs GmbH, Frankfurt am Main, Germany), and the accuracy of sequences was ascertained by Sanger sequencing [47]. Clones with seven (TA)-repeats in the TRD-encoding region of M2 gene region were chosen for the further analysis, expressing rM2 in full length (without a TA-based frame shift). As shown in Supplementary Figure S2, the IPTG-induced expression of His_6_-tagged proteins was documented in immunostaining, as formerly published [48].

**Figure 4:**
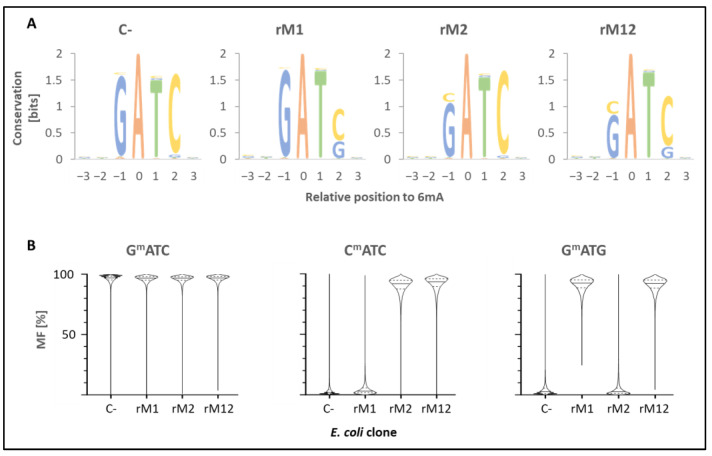
**Methylation motifs of *E. coli* clones.** Total DNA of *E. coli* DH5αF’IQ-clones, expressing MTase parts M1 or M2 or the fusion-MTase M12, was sequenced by ONT and the methylation status analysed by dorado. The plasmid-free *E. coli* was used as a negative control (C-). (**A**) Sequence logos (+/− 3 nt) of 6mA methylations (position 0). (**B**) Violine plots of the 6mA methylation frequencies (MF) in the different motifs GATC, GATG and CATC.

The total DNA of the *E. coli* clones, expressing rM1, rM2 or rM12, was sequenced by ONT, and methylation scores were calculated with dorado using the sequences of the *E. coli* DH5aF’IQ genome, F’-Plasmid and respective pQE30-plasmids as references. In comparing the methylation profile of the *E. coli* clones with and without recombinant MTase rM1, rM2 or rM12 expression, 4mC and 5mC methylations (other than the chromosomal C^m^CWGG) were not detected, but 6mA methylation was (see Figure 4).

Building seven nucleotide-sequence logos with the methylated adenine in position 0, it became obvious that 6mA was always followed by T (Figure 4A), and the (−1) and (+2)-positions consisted of G and/or C. Due to a *dam* presence in the *E. coli* DH5aF’IQ genome, G^m^ATC was present in all tested clones and was the one and only 6mA-methylation found in the plasmid-free *E. coli* (C-). Beside G^m^ATC, G^m^ATG was detected in the rM1- and C^m^ATC in the rM2-expressing clone. As shown in Figure 4B, methylation levels at the complementary motif remained close to control values. Although a Wilcoxon matched-pairs signed-rank test indicated a statistically significant increase (*p* < 0.0001) relative to the control, this result was interpreted as an effect of the large number of genomic positions analyzed (*n* = 26,159) rather than a biologically meaningful effect. The minimal differences in median methylation frequency therefore indicate the absence of substantial off-target methylation. In the rM12-expressing *E. coli*, G^m^ATG and C^m^ATC motifs were detected in significant amounts (Figure 4B).

### 2.4. Methylation-Sensitive Restrictions Support the MTases Specificity

**Figure 5:**
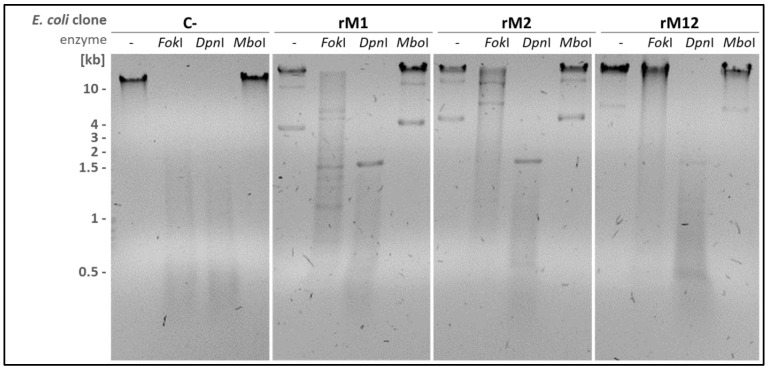
**Methylation-sensitive restriction of genomic DNA of MTase-expressing *E. coli* clones.** Genomic DNA of *E. coli* DH5α F’IQ-clones (0.75 µg DNA/lane), expressing MTase parts M1 or M2 or the fusion-MTase M12, was restricted with *Fok*I (GGATC(N_9/13_)), *Dpn*I (G^m^A/TC) or *Mbo*I (N/GATC). The plasmid-free *E. coli* was used as a negative control (C-). Restricted DNA was separated on 1% (*w*/*v*) agarose gel beside an unrestricted sample (-).

In an MSR analysis, restriction enzymes *Dpn*I and *Mbo*I were used to control *dam* G^m^ATC methylation of the *E. coli* DNAs. As expected, all DNA samples were restricted by *Dpn*I (but not *Mbo*I), as *Dpn*I needs a 6mA methylation of the motif for cutting, which is inhibitory for *Mbo*I restriction (Figure 5).

Restriction enzyme *Fok*I, which cuts the 6mA-unmethylated motif 5′-GG**A**TG(N_9/13_), was used to prove GATG/CATC as the target motif of M12.*Mho*VII. DNA of the plasmid-free *E. coli* was completely restricted by *Fok*I, which gave evidence of the unmethylated motif. An increased protection of the DNA was observed in *E. coli* clones expressing either rM1 or rM2 to rM12, demonstrating that both MTase parts, M1 and M2, were essential for the complete protection of the DNA.

### 2.5. M12.MhoVII Activity Is Not Constitutively Expressed in the Native Host

**Figure 6:**
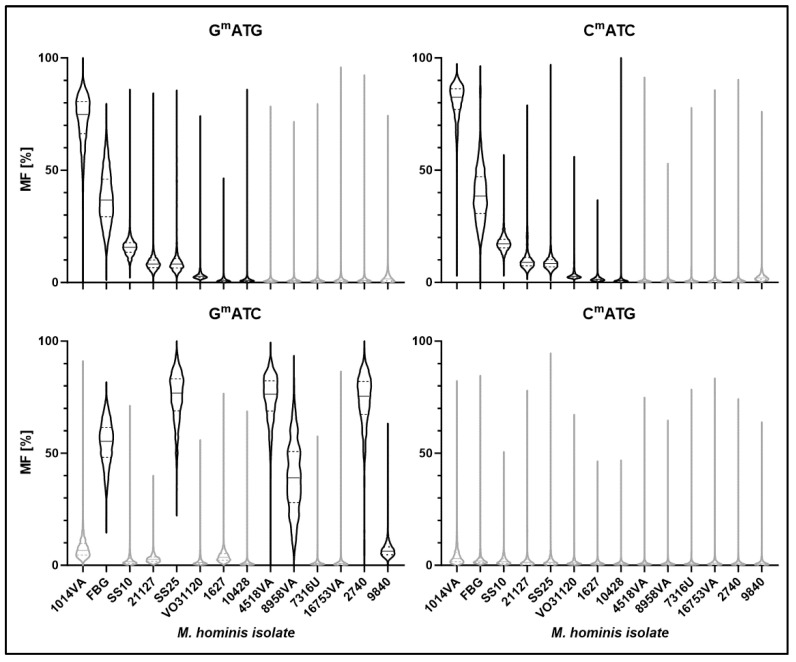
***Mho*VII-specific methylations in *M. hominis* isolates.** Genomic DNA of *Mho*VII-positive and -negative *M. hominis* strains was sequenced by ONT, and the methylation status analysed by dorado. Violine plots demonstrate the 6mA methylation frequencies (MF) in the *Mho*VII-specific motifs GATG and CATC, with GATC (*dam*) and CATG as controls. Black and grey plots indicate the proven present and absent or yet-undetected MTase genes, respectively.

ONT-sequenced genomes of *Mho*VII-positive and -negative *M. hominis* strains were bioinformatically analyzed for G^m^ATG/C^m^ATC-methylations. The calculation of G^m^ATC (*dam*2) served as a control and of C^m^ATG to complete all C/G variations (Figure 6).

6mA methylation analysis was not only restricted to GATG and CATC, but also GATC and CATG motifs to exclude potential signal interference from unrelated MTase activities within the same isolate. To ensure reliable results, the methylation frequencies of 6mA-modified adenines were calculated only for positions with a sequencing coverage of ≥30. Thus, only a minimal proportion of total G^m^ATG or C^m^ATC sites was excluded by this filtering step, as nearly all retained positions exceeded a coverage of 300× (see Supplementary Figure S9). *Dam*-specific G^m^ATC methylation generally corresponded to the presence of the *dam* gene in strains FBG, SS25, 4518, 8958VA and 2740. The reduced methylation frequency in strains FBG and 9840 correlated with the truncation of the 3′-end of *dam* in strain FBG and 9840, but was unexpected in strain 8958 that carried the complete *dam*2 RM system [8]. 6mA methylation of CATG was found in neither *Mho*VII-positive nor -negative strains. As expected, no 6mA methylation of GATG/CATC was detected in *Mho*VII-negative strains, while the frequency in *Mho*VII-positive strains varied between 11.4% in isolate SS25 and 76% in isolate 1014VA. Low-level background methylation (<5%) observed in strains VO31120, 1627, and 10,428 was interpreted as methodological background level off false-positives by ONT sequencing.

### 2.6. Recombinant R.MhoVII Cleaves Only DNA with 6mA-Unmethylated GATG/CATC Sites

For a comprehensive characterisation of *Mho*VII activity, purified rR.*Mho*VII was intended for use in MSR analysis. To ensure compatibility with downstream restriction reactions, buffer conditions during cell disruption and purification were kept as close as possible to the rCutSmart buffer, resulting in the use of a Tris-based buffer system and the avoidance of glycerol and β-mercaptoethanol. Despite further optimization attempts, including reduced expression temperature and cell lysis on ice, a substantial fraction of the protein remained insoluble and exhibited poor binding to the Ni-NTA resin (Supplementary Figure S4). However, purified amounts of rR.*Mho*VII were obtained for restriction assays (see Western blot analysis (Supplementary Figure S3).

**Figure 7:**
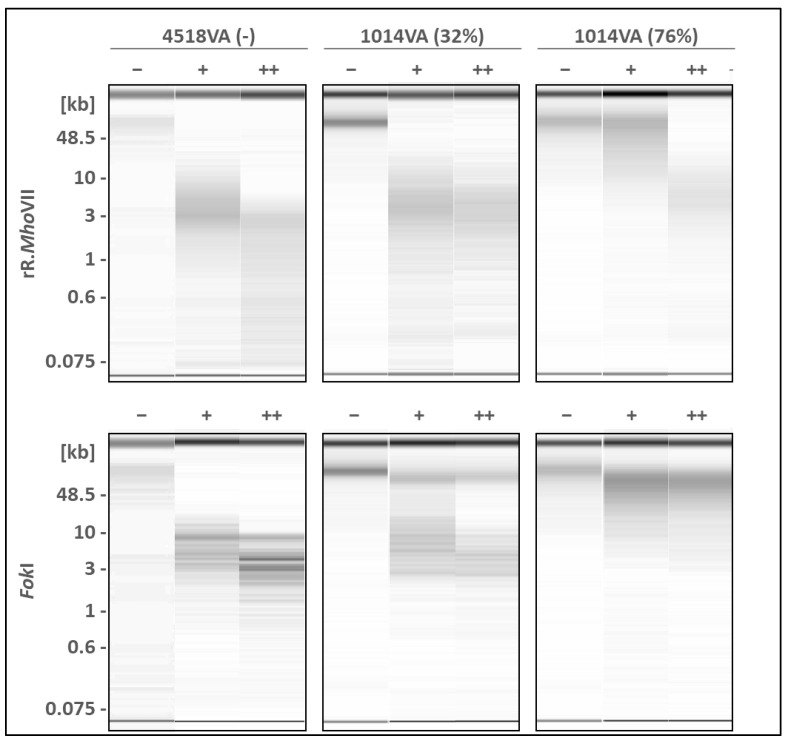
**MSR analysis of *M. hominis* DNA.** Genomic DNA of *M. hominis* isolates 4518VA (6mA unmethylated GATG/CATC) and 1014VA (with two preparations of 32% and 76% MF of G^m^ATG/C^m^ATG) was incubated for 2 h at 37 °C with recombinant rR.*Mho*VII or *Fok*I (as a control) in increasing amounts (−/+/++; as specified in Section 5.). Restriction patterns were visualized by capillary electrophoresis.

Genomic DNA of the isolates 1014VA and 4518VA was used as a DNA source as representatives for *mho*VII presence and absence, respectively. Two DNA preparations of the isolate 1014VA were analysed, as they differed in the methylation frequency of GATG/CATC (32% and 76% MF, as calculated by ONT). Isolate 4518VA was used as a negative control because it lacks the RM system and had been shown in *Fok*I restriction to be GATG/CATC unmethylated. As to be expected in case of a GATC/CATC-targeting, R.*Mho*VII cut the unmethylated DNA of 4518VA (Figure 7).

With increasing amounts of rR.*Mho*VII, there was an elevated restriction of the 6mA GATG/CATC-unmethylated DNA of isolate 4518VA. Restriction of the *Mho*VII positive isolate 1014VA was inhibited, with a stronger effect the lower the rR.*Mho*VII amount and the higher the methylation frequency of G^m^ATG/C^m^ATC (Figure 7, 1014VA (MF 76%)). A comparable shift in restriction patterns was observed when using *Fok*I, which targets and cuts the same unmethylated 6mA motif with an additional G at the 5′ end (GGATG/CATCC). The resulting lower number of *Fok*I to R.*Mho*VII restriction sites per genome led to larger fragments overall for *Fok*I.

These restriction data demonstrated that unmethylated GATG/CATC motifs are the target sites of the restriction endonuclease R.*Mho*VII. Fragmented genomic DNA from *M. hominis* isolate 4518VA was subsequently analysed by Oxford Nanopore sequencing to gain insights into the enzyme’s cleavage site. Distances between the nearest GATG/CATC motif and the read termini were calculated to infer regions where DNA fragments preferentially ended and, consequently, the likely restriction site. However, this approach did not allow the identification of a defined cleavage site in the immediate proximity of the methylation motif (Supplementary Figure S10). No significant enrichment of read ends at specific distances relative to the motif was observed.

### 2.7. RM.MhoVII Transcript Levels Are Decreased in Chronic Stage of Hela Infection

**Figure 8:**
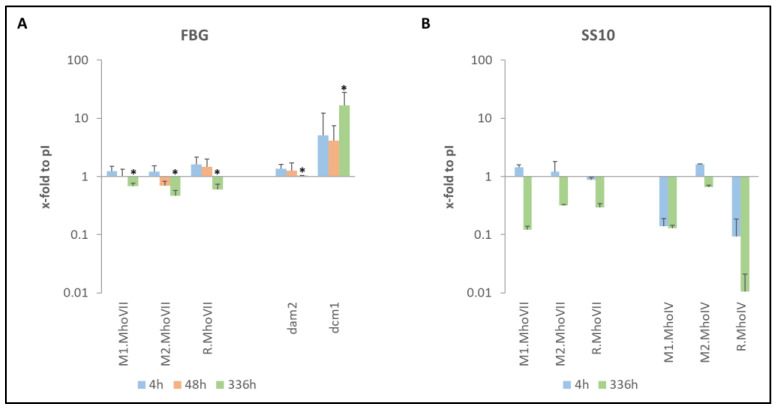
**Transcript levels of selected REases and MTases during HeLa infection.** Total RNA from HeLa cells infected with *M. hominis* isolates FBG and SS10 for 4 h, 48 h or 336 h was analysed by RT-qPCR. Transcript changes were calculated relative to the expression at the start of infection (1 h pI) and normalized to the reference genes *lgt* and *gap* (as described formerly [49]). (**A**) Mean transcript levels of *mho*VII genes and the solitary MTases *dam*2 and *dcm*1 in isolate FBG were determined from four or two (*) biological replicates, measured in duplicates. (**B**) Transcript levels of *mho*VII genes and *mho*IV genes were quantified for isolate SS10 at 4 h and 336 h pI. (Due to a degradation, RNA of time point 48 h could not be analysed for isolate SS10.).

As bacterial DNA methylation is a known key regulator of gene expression, thus influencing virulence factors and the ability of bacterial biofilm formation, we next analysed changes in the *mho*VII transcription in HeLa cell infection [49]. Since the RNA samples originated from previous in vitro HeLa cell infection assays (2015–2017), RNA quality was firstly re-evaluated by fragment analysis, and the synthesized cDNA was then controlled by verifying published transcription levels of gene MHO_2080 (see Supplementary Figure S7) [49]. As shown in Figure 8, *mho*VII transcripts of *M. hominis* strains FBG and SS10 were normalized to transcript levels of the reference genes *lgt* and *gap* at 1 h post infection (pI).

Over the course of infection, *mho*VII transcript levels were initially (4 h pI) slightly higher than those of the reference genes but displayed a tendency to decline up to the chronic infection stage (336 h pI) in isolate FBG. Similar transcript profiles were observed for *dam*2, which encodes a truncated but still active MTase responsible for G^m^ATC methylation (Figure 8A). In contrast, *dcm*1, encoding the solitary MTase responsible for C^m^CWGG methylation, showed the highest expression (5–30-fold increase) at 336 h pI, representing the chronic infection stage. Although the RNA preparation of isolate SS10 at 48 h pI no longer met quality standards and was omitted from analysis, comparable expression behaviour was observed; the expression of *Mho*VII (and *Mho*VI as well) decreased significantly from the onset of infection to the chronically infected culture. This led to the thesis that these RM systems must be switched off for mycoplasma survival in chronically infected hosts.

## 3. Discussion

Nowadays, next-generation whole genome sequencing allows the direct identification of 6mA, 5mC and 4mC DNA methylations without relying on bisulfite-treated DNA or methylation-sensitive restriction [50,51,52]. Although PacBio single-molecule real-time sequencing historically served as the gold standard, rapid improvements in Oxford Nanopore Technology (ONT), including enhanced base-calling models, improved chemistry, and further developed R10 flow cells, have closed the performance gap, enabling robust and accurate methylation analysis combined with comparably low cost [53,54,55]. The user-friendly technology is now the basis for a variety of customer-developed tools that are tailored on the specific and diverse requirements of modern sequence-based research and diagnostics [56,57,58].

In this study, Oxford Nanopore sequencing yielded a high sequencing depth (>300×; see Supplementary Figure S9), enabling the robust and direct detection of methylated motifs generated by recombinant *Mho*VII MTases. These results were proven by methylation-sensitive restriction assays. Although REBASE lists MTases that are predicted to be associated with GATG/CATC methylation, these annotations were not supported by experimental evidence [18]. To our knowledge, this study therefore provides the first experimental validation of a restriction–modification system that methylates the non-palindromic GATG/CATC motif. It was demonstrated that the *Mho*VII system of *M. hominis* comprises two fused, 6mA-specific MTase domains together with an active restriction endonuclease that selectively cleaves unmethylated GATG/CATC sites, thereby proving both restriction and modification activities.

In addition to methylation detection, Oxford Nanopore sequencing was explored as an approach to approximate the cleavage position of rR.*Mho*VII by analyzing the distribution of read termini relative to the GATG/CATC recognition motif. However, this analysis did not reveal an enrichment of read ends in the proximity of the motif and therefore did not allow the definition of a specific restriction site. Several technical factors likely limit the suitability of ONT data for this purpose. Ligation-based library preparation involves end-repair and dA-tailing, which modify native DNA termini prior to adapter ligation and can obscure true cleavage positions [59]. In addition, base-calling accuracy is reduced toward read ends, and alignment-related trimming or soft clipping further shifts the apparent read termini away from the original cut site. An alternative explanation is that R.*Mho*VII may not cleave DNA at a single, fixed position relative to its recognition motif. To resolve this question, future experiments will employ run-off sequencing of defined PCR substrates cleaved with freshly purified R.*Mho*VII followed by Sanger sequencing, which allows the precise mapping of strand cleavage positions at single-nucleotide resolution [60].

The nuclease activity of the purified recombinant rR.*Mho*VII was proved by restriction assays. Of note, the heterologous expression of the restriction enzyme did not impair the viability of *E. coli* (through self-cleavage), although protective methylation appeared to be absent. The most likely reason for this was the expression of the recombinant REase in inclusion bodies and the resulting failure for proper folding, which is necessary for activity. This is in line with our findings, as rR.*Mho*VII was insoluble under native conditions and needed the addition of the detergent n-lauryl sarcosine (NLS) for solubility. If the dilution of rR.*Mho*VII samples resulted in N-LS values below the micelle concentration, a band with a higher molecular weight was detected in the Western blot alongside the monomer, which could correspond to the dimer (see Supplementary Figures S3 and S4). The Type IIS restriction enzymes *Alw*I and *Fok*I are known to require dimerization for double-strand cleavage [26,61]. Although no catalytic core motif similar to that of *Fok*I could be identified in R.*Mho*VII, helical domains similar to α helices 4 and 5 of *Fok*I, which are essential for dimerization, were present (see Supplementary Figure S5).

The MTases of the *Mho*VII were unmasked in this study to methylate GATG/CATC, a non-palindromic recognition motif that has been virtually unknown in the literature to date. In general, recognition motifs comprising four base pairs occur more frequently in Type IIP restriction enzymes, which often act as monomers, while non-palindromic motifs with five to six base pairs were characteristics of Type IIS restriction enzymes, such as *Fok*I or *Hga*I, which need a dimerization for cleaving both strands [24,25,26,27,62]. This suggests that *Mho*VII belongs to the Type IIS subgroup. To date, REBASE lists 530 different motifs that were assigned to a Type IIS RM system, and only two of them are four base pairs long [18]. Only five MTases that were assigned to GATG/CATC motifs are listed in REBASE, but none of them are supported by published observations. The supposed CATC-specific MTases should encode solitary MTases of γ-group (methyl-donor domain (MD)-catalytic domain (CA)-target recognition domain (TRD)), thus differing in domain order to the β-group (CD-TRD-MD) of M1.*Mho*VII and a-group (MD-TRD-CD) of M2.*Mho*VII [18]. Nevertheless, they still clustered within the M1.*Mho*VII, branch suggesting a common origin (see Supplementary Figure S6).

According to BLAST analysis, the M1.*Mho*VII MTase was assigned to the N4-N6 MTases family, but 4mC methylation was considered unlikely in advance due to the identification of typical 6mA MTase domain features (e.g., a DPPY domain instead of SPPY in the CM-II domain) [63]. The experimental data of this study ultimately confirmed 6mA methylation activity, which is notable because 6mA methylation seemed to be underrepresented in *M. hominis*. In *Mycoplasma agalactiae,* Type II RM systems were shown to be distributed almost equally between 5mC, 6mA and even 4mC methylation, but in *M. hominis,* 11 5mC MTases were detected compared to only four 6mA and one 4mC MTases [8,46,64]. Nine of them have experimentally confirmed recognition motifs, including the RM system *Mho*VI, that recognizes a non-palindromic sequence motif and thus also belongs to the Type IIS group. *Mho*VI expresses two separate MTases and, even though transcript levels were comparable to *Mho*VII, the methylation activity of *Mho*VI was always high (>85%) in all tested isolates, whereas *Mho*VII activity was shown in this study to differ heavily between isolates and individual cultures. On the other side, *Mho*VII was twice as common (27.6%) as *Mho*VI (13%) among the *M. hominis* isolates tested [46]. Comparable data from *Helicobacter pylori* has led to the hypothesis that recently acquired strain-specific RM systems tend to remain active, whereas conserved systems that have been widespread in various isolates for a long time more often lose activity due to mutation [65]. The combination of a lower methylation activity but higher prevalence therefore suggests that *Mho*VII was acquired earlier in the evolutionary history of *M. hominis* than *Mho*VI.

The regulation of RM systems can be quite complex, involving multiple intrinsic promoters and terminators and additional transcriptional regulator proteins, with C-proteins being the most prevalent and most studied regulators [41,66,67,68]. Methylation can still have a large downstream effect on gene expression by controlling regulatory genes far away from the methyltransferase [69]. Many RM systems are more easily regulated through a polycistronic organization, to which *Mho*VII seems to belong [44,70]. The *Mho*VII gene cassette was shown to express a polycistronic mRNA and lacks 1. additional genes (putatively encoding regulatory elements), 2. active internal promoter/terminator sites and 3. *Mho*VII recognition motifs in the promoter region that are often used for self-regulation of MTases through methylation [71,72]. However, differences in the *Mho*VII MTase activity were observed between isolates and also in biological replicates despite constant transcription levels. The factors that are responsible for such differences between cultures of the same isolate are unknown. The differences in methylation frequencies between different isolates may derive from genomic heterogeneity. A variety of Mobile Genetic Elements, which are proposed to interact with RM systems, might influence *Mho*VII activity [7,8,73,74]. The observations of this study mirror results from previous studies of *Mho*VI, Dam1/2 and Dcm1 in *M. hominis*, which also found no correlation between methylation activity and transcript levels [8,46], and indicate a post transcriptional regulation in the mid-to-late logarithmic growth phase of *M. hominis,* whose mechanism we have not yet discovered.

Remarkably, a regulation of *Mho*VII expression on the transcript level was observed in *M. hominis* infection’s of HeLa cells. After 4 h, post-infection (pI) transcript levels *Mho*VII were slightly elevated compared to the reference genes at 4 h pI, but they declined over course of infection and were downregulated in long-term infection (336 h pI). Solitary MTases and RM systems are known to interplay with cellular mechanisms, thus influencing virulence and pathogenicity [30,36,43,75]. While the downregulation of a solitary MTase’s activity was shown for *Mycoplasma hyorhinis* at CpG sites during infection, GAT^m^C methylation remained high throughout infection [76]. In *M. hominis*, it was shown that clinical isolates have an increased abundance of Type I and Type III RM system components compared to laboratory strains, so it was suggested that RM systems influence virulence [77]. A former study using a customized *M. hominis* microarray has shown that infection leads to the differential expression of a large subset of genes involved in almost all cellular processes of *M. hominis* [49]. Unfortunately, the microarray was based on less virulent genomes of PG21 and LBD4, with the result of an underrepresentation of RM systems. This study demonstrates differential transcription during infection of all Type II MTase/REase genes known to be present in the *M. hominis* isolates FBG and SS10. *Mho*VII was downregulated during infection, as well as the *Mho*IV RM system. While RM systems were downregulated as the infection progressed, the solitary MTase Dcm1 showed highly increased transcription levels in isolate FBG, suggesting that this MTase activity is rather relevant for the persistence of *M. hominis* infection [8]. An increased Dcm methylation (C^m^CWGG) associated with an increase in virulence was already demonstrated for *Porphyromonas gingivalis* and *E. coli* [30,66]. In the invasive *M. hyorhinis*, MTases were described to be able to translocate into the human host cell nucleus and to methylate the genome, indicating a potential mechanism of pathogen–host interaction and host cell regulation [76]. However, *M. hominis* mainly colonizes the surface of urogenital cells [78,79]. Thus, the downregulation of RM systems might help *M. hominis* to adapt to its host without triggering its defensive mechanisms.

The results of this study point out that the *Mho*VII expression is affected by posttranscriptional mechanisms (as demonstrated in axenic cultures) and a transcriptional regulation (as detected in infections). Another possible regulatory option was not addressed in this study: the AT-repeat region in the M2-part of the RM system, which suggests a regulation through phase variation. Type II RM systems are widespread in bacterial pathogens, where they modulate genome methylation, often influenced by phase variation through simple sequence repeats (SSRs) [23]. This allows the pathogen to produce diverse phenotypes in order to better adapt to specific environments and hosts during infection [80]. A regulation of MTase expression through phase variation was demonstrated in *H. pylori* or *Campylobacter jejuni* [65,81,82]. In other bacteria incl. mycoplasma, SSRs are capable of changing the expression or specification of MTases [83]. In *M. agalactiae,* it was shown that a poly GA repeat in two Type III MTases was used to induce phase variation and alter the methylation status of the genome [64]. Thus, the TA-repeat in M2.*Mho*VII may also be the target for phase variation of the *Mho*VII MTase and thus represent the third level of *Mho*VII regulation and a possible explanation for the differences in methylation activity.

Most commercially available Type II restriction enzymes typically recognize palindromic sequences of 4–6 bp length and cut within or near the recognition motif. They are widely used in molecular biology today [22,84]. Type IIS restriction endonucleases are increasingly applied in molecular biology and biotechnology because they cleave outside their recognition motif and generate specific overhangs essential for modular cloning systems, such as Golden Gate assembly [85]. The characterization of new RM systems such as *Mho*VII is therefore not only important for understanding bacterial defense biology but also relevant for expanding the molecular tools with enzymes of novel specificities.

## 4. Conclusions

*Mho*VII, a novel restriction–modification system of *M. hominis,* was characterized in this study. It consists of two distinct fused methyltransferases that target and 6mA-methylate the non-palindromic motif GATG/CATC, which protects the host DNA from cleavage by the associated restriction endonuclease. The non-palindromic motif suggests a classification of the restriction endonuclease as Type IIS, as it recognized asymmetric DNA sequences. However, the final evidence—that it also cleaves the DNA outside of its recognition sequence—is still missing. *Mho*VII activity was context-dependent, suggesting post-transcriptional control in axenic cultures and transcriptional downregulation during HeLa cell infection. Future studies based on these results should not only focus on the identification of factors that influence the methylation activity and characterization of its function in *M. hominis* virulence, but also include the identification of the cutting sites of R.*Mho*VII, as this newly identified recognition motif may also offer potential applications in molecular biology and genome engineering.

## 5. Materials and Methods

### 5.1. Quantitative PCR

The qPCR assays were carried out in a total volume of 25 µL consisting of 1× Takyon SYBR MasterMix dTTP Blue with fluorescein (Eurogentec, Seraing, Belgium), 300 nM of each primer and 2.5 µL of genomic DNA (1 ng/µL) or cDNA (0.8 ng/µL) solution, which was derived from 100 ng RNA. PCR assays for the construction of expression plasmids were carried out in a total volume of 25 μL consisting of 1× SuperMix (Biorad, Hercules, CA, USA), 300 nM of each primer and 2.5 μL of genomic DNA (10 ng/µL), which was prepared as formerly published [9]. Oligonucleotides (primers) were designed using Applied Biosystems Primer Express Software v3.0.1 (Thermo Fisher Scientific, Waltham, MA, USA; for TaqMan-PCR) or PrimerSelect of DNASTAR (Madison, WI, USA; for conventional PCR) and ordered from metabion (metabion, Planegg/Steinkirchen, Germany). Primer sequences used for expression plasmid construction are listed in Supplementary Table S1.

Thermal cycling conditions for transcript quantification using Taq-primers were as follows: 1 cycle at 95 °C for 5 min, followed by 35 cycles of 95 °C for 30 s and 60 °C for 30 s. The PCR conditions for the detection of overlapping transcripts were as follows: 1 cycle at 95 °C for 5 min followed by 35 cycles of 95 °C for 30 s, 54–64 °C (depending on the primers’ annealing temperature) for 30 s, and 72 °C for 1 min. Fragment V was used for the normalization of PCR products, as it is located within the M12.*Mho*VII open reading frame and does not contain any predicted regulatory elements.

For expression plasmid construction, overlapping fragments were fused by SOE (splicing by overlap extension)-PCR as published [86]. Briefly, PCR fragments were assembled into a single full-length gene by designing overlapping regions between adjacent fragments. These overlaps enabled the first step, the primerless extension and fusion of the overlapping fragments, followed by the amplification of the assembled gene using external primers (Scheme is shown in Supplementary Figure S8).

### 5.2. Cloning of mhoVII

The PCR products, comprising the MTases gene region M1 (formerly named *dcm*8) or M2 (*dam*3) of isolate SS10, were cloned into *Bam*HI-*Hin*DIII sites of pQE30 (Qiagen, Hilden, Germany). To generate an M1-M2 fusion (named M12), the M1 region was reamplified with the primer pair M1-F1_BamHI and M1-R6_*Bam*HI and cloned into the *Bam*HI-site of the M2-plasmid. Plasmids were propagated into *E. coli* Dh5αF’IQ (New England Biolabs GmbH (NEB), Frankfurt am Main, Germany) and cultivated in LB medium supplemented with 100 mg/L ampicillin (LB-Amp). A pCola-Duett-1-construct expressing the His-tagged restriction endonuclease rR.*Mho*VII was ordered from GenScript (GenScript, Rijswijk, The Netherlands), propagated into *E. coli* BL21 D3 (NEB) and cultivated in LB-medium with 100 mg/L kanamycin (LB-Kan). The inserts of selected clones were checked for accuracy using Sanger sequencing [47].

### 5.3. Expression of MhoVII Proteins

The heterologous expression of His_6_-tagged proteins, induced by 0.02mM isopropyl-β-D-1-thiogalactopyranoside (IPTG), was documented in His_4_-immunostaining as formerly published [48]. For rR.*Mho*VII preparation, the cells of 15 mL *E. coli* BL21-pCola-Duett-1_rR.*Mho*VII culture were sedimented, resuspended in 1 mL Tris sonication buffer (TSB) [20 mM Tris/HCl, 500 mM KCl, pH 8.0] supplemented with 5% (*w*/*v*) n-lauryl sarcosine (N-LS), 4 mg/mL lysozyme, incubated for 2 h on ice and then sonicated (2 min interval and 1.5 min constant). The cleared lysate was 20-fold diluted in TSB and incubated with Ni-NTA (150 µL 50% (*w*/*v*) slurry in TBS—0.25% N-LS) for 3 h at RT. Ni-NTA was washed four times with TSB containing descending amounts of N-LS; rR.*Mho*VII was eluted in N-LS-free TSB, pH 4.0.

### 5.4. Bacterial Culturing, Protein and Nucleic Acid Preparations

*M. hominis* isolates, which derived from the internal culture collection of the institute, were cultivated overnight at 37 °C in arginine medium (10% horse serum, 2.1% PPLO broth, 1% yeast extract, 1% L-arginine, 0.002% phenol red, pH 6.5) without shaking under anaerobic conditions until they reached the mid- to late-logarithmic growth phase [46]. Genomic DNA and total RNA from *E. coli* (3 mL) and *M. hominis* (50 mL) cultures were prepared as described in detail previously [46,78]. The DNA concentration was measured by Invitrogen Qubit 4 Fluorometer Qubit (Invitrogen, Carlsbad, CA, USA); the RNA concentration and the quality of RNA and DNA were verified by NanoDrop 1000 Spectrophotometer (Thermo Fisher Scientific, Waltham, MA, USA).

### 5.5. Methylation-Sensitive Restriction (MSR) Analysis

A total of 0.75 µg of *E. coli* DNA was digested with 3 U restriction enzymes *Fok*I, *Mbo*I or *Dpn*I (NEB, Frankfurt, Germany) with 1x rCutSmart buffer (NEB, Frankfurt, Germany) in a total volume of 15 µL at 37 °C for 1 h, followed by heat inactivation at 80 °C for 20 min [8,87,88]. To estimate R.*Mho*VII activity, 14 ng DNA aliquots of *M. hominis* strains were restricted with 0.081 U *Fok*I/µg DNA and 0.9 U *Fok*I/µg DNA, or 10 ng or 30 ng rR.*Mho*VII in rCutSmart buffer (NEB, Frankfurt, Germany), in a total volume of 5 µL for 2 h at 37 °C. Restriction patterns were analysed via Agilent Fragment Analyzer system DNF-464-33—HS Large Fragment 50Kb (Agilent Technologies, Santa Clara, CA, USA) with 3 ng restricted DNA at the *Genomics & Transcriptomics Laboratory* of the Biological Medical Research Centre of the Heinrich-Heine University of Duesseldorf. MSR analysis was performed in technical duplicates.

### 5.6. Oxford Nanopore Sequencing and Methylation Analysis

The whole-genome sequencing of the *E. coli* clones was performed using the native barcoding kit V14 (SQK-NBD114.24) from Oxford Nanopore technology (Oxford Nanopore Technologies, Oxford, UK) according to the manufacturer’s instructions. The DNA libraries were loaded on PromethION R10.4.1 flow cells and sequenced on the PromethION PC24 with the MinKNOW v24.06.10 software. Using a custom script in Python v3.9.17, read IDs from the barcoded FASTQ files were extracted and used for the classification of unsorted POD5 reads. Methylation scores for 4-methylcytosine (4mC), 5-methylcytosine (5mC) or 6-methyladenine (6mA) were computed via the basecaller Dorado (v0.7.3); https://github.com/nanoporetech/dorado (accessed on 28 February 2025)) on *Escherichia coli* strain NEB5-αF’IQ genome (CP053607.1), F’-plasmid (CP053606.1) and pQE30-rM1+/−rM2 plasmid sequences, employing the base calling models dna_r10.4.1_e8.2_400bps_sup@v5.0.0_4mC_5mC@v1 and dna_r10.4.1_e8.2_400bps_sup@v5.0.0_6mA@v1. The conversion of BAM output-files to BED files was done using the modkit tool (v0.2.7). Methylation scores were calculated as follows: fraction_modified [%] = (N_mod_/(N_mod_ + N_canonical_ + N_other_mod_ + N_diff_)) × 100. Scores with a coverage of <30 were filtered out. The positions of methylations and putative sequence motifs were identified in genomes with Python v3.9.17 using customized scripts. DNA fragments, which derived from the restriction assay of *M. hominis* 4518VA DNA with rR.*Mho*VII, were sequenced on FLO-PRO114M flow cells using the SQK-NBD114-24 kit (Oxford Nanopore Technologies, Oxford, UK).

### 5.7. Hela Cell Infection Assay

The human cervical carcinoma cell line HeLa S3 (ATCC CCL-2.2) that was uninfected or infected by *M. hominis* strains FBG or SS10 was cultivated in DMEM, and 10 million cells per 75 cm^2^ cell culture flask were infected with 50 MOI (multiplicity of infection) of the respective *M. hominis* isolate for 1 h, 4 h, 48 h and 336 h, as published before [49]. The experiment was performed in duplicates for each time point. For FBG infection, two to four biological replicates and one assay for SS10 infection were used. Total RNA was purified from each infection assay; RNA from time point 1 h of infection was used as a reference.

### 5.8. Bioinformatic Analysis

Procaryotic promotor and terminator sites were predicted with ProPr v2.0 and inverted repeated /stem loops with EMBOSS: palindrome (https://www.bioinformatics.nl/cgi-bin/emboss/palindrome (accessed on 9 September 2025). Multiple sequence alignments were calculated using Geneious Pro v5.5.8 (Dotmatics, Boston, Madison, WI, USA). MegAlign v5.08 of the Lasergene software package (DNAStar, Madison, WI, USA) was used with default settings for phylogenetic tree construction. The REBASE database-driven analysis of MTase presence and target motifs was performed with MPore software developed by Azlan Nizar (https://github.com/DiltheyLab/MPore (accessed on 27 October 2025)). To predict secondary structures in protein sequences, the Phyre2.2 web tool (https://www.sbg.bio.ic.ac.uk/phyre2/html/page.cgi?id=index (accessed on 18 November 2025)) was used.

The analysis of rR.*Mho*VII restriction sites was performed by aligning all reads to the reference using Minimap2 v.2.17 with default parameters. The alignment terminus for each read was extracted when it was detected within a 20 bp window around GATG or CATC motifs, using customized scripts in Python v3.12.3. Distances, referring to the position of the last aligned base, were calculated relative to the adenine within each motif.

## Figures and Tables

**Table 1:** 6mA MTase types in MTase homologs of *Mho*VII.

Organism	Code	Acc-No MTase	AA	(1.1) YhdJ (M1.*Mho*VII) CDD:440623	(1.2) MethyltransfD12 CDD:451538	(2.1) DNA Adenine Methylase CDD:442619	(2.2) Dam (M2.*Mho*VII) CDD:440107	Code	Acc-No Rease	AA	^1^ RE_AlwI CDD:401443 ^2^ PDDEXK Family Nucleases CDD:477358
*M. hominis*	Mho	WP_175934170	534	7..238	237..517			MhoR	WP_175950169.1	524	
*M. pirum*	Mpi	WP_081794293	531	16..239	242..474			MpiR	WP_027124015.1	575	
* M. caviae *	Mca	WP_218017466	71	9..>42							
* M. caviae *	Mca	WP_218017467	209	<1..117	130..>187			McaR	WP_126118161.1	529	
*M. salivarium*	Msa	WP_236148935	550	26..248	260..547			MsaR	WP_236148934.1	525	(^1^ 1..525)
*M. hyosynoviae*	Mhy	WP_273570538	534	9..231	243..530			MhyR	WP_036444484.1	507	
*M. mucosicanis*	Mmu	WP_170218490	524	5..226	234..521			MmuR	WP_141483791.1	515	
*C. M. glomeromycotorum*	Mgl	MCE8162666	753	228..452		459..753		MglR	MCE8162667	535	(^1^ 1..535)
*M. meleagridis*	Mme	OAD18378	642			16..338	342..639	MmeR	-	-	
*M. agalactiae*	Mag	WP_233736886	313				11..308				
*M. agalactiae*	Mag	WP_233736887	365			38..361		MagR	WP_233736888.1	677	^1^ <348..638
*M. bovis*	Mbo	WP_013456178	365			38..361		MboR	WP_013456255.1	677	^1^ <348..638
*M. bovis*	Mbo	WP_013456250	313				12..308				
*M. mycoides*	Mmy	WP_020862963	362			1..362		MmyR	WP_020862964.1	679	^1^ 1-679 ^2^ 351..679
*M. mycoides*	Mmy	WP_129868728	301				7..299				
*M. agalactiae*	Mag	MCE6061993	365			1..365		Mag			
*S. uberis*	Sub	WP_154631601.1	709			75..399	409..707	SubR	WP_154631600.1	678	^1^ 1-678 ^2^ 343..677
*L. phage AM1*	Lph	YP_009904999.1	227	7..227				LphR	-	-	

^1^ RE_AlwI CDD:401443 ^2^ Superfamily of PDDEXK nucleases including very short patch repair (Vsr) endonucleases, archaeal Holliday junction resolvases, MutH methyl-directed DNA mismatch-repair endonucleases, and catalytic domains of many restriction endonucleases, such as *Eco*RI, *Bam*HI, and *Fok*I.

## Data Availability

Genome and protein sequences were obtained from the National Library of Medicine from the National Center for Biotechnology Information. The corresponding accession numbers are provided in the text. Normalized genome sequences from Nanopore assemblies of genomes of clinical *M. hominis* strains are available at OSF [https://osf.io/gfbzv/overview?view_only=5e305a51af1f4fb6a9cdc2787b755e66, accessed on 16 December 2025]. Nanopore raw data will be provided on request.

## Supplementary Materials

The following supporting information can be downloaded at: https://www.mdpi.com/article/10.3390/ijms27031591/s1.

## Abbreviations

The following abbreviations are used in this manuscript:

Dam	DNA-adenine-methyltransferase
Dcm	DNA-cytosine-methyltransferase
MF	Methylation frequency
MSR	Methylation-sensitive restriction
MTase	DNA-methyltransferase
ONT	Oxford Nanopore Technology
REase	Restriction endonuclease
RM	Restriction–modification
SSR	Simple sequence repeat
TRD	Target recognition domain
CD	Catalytic domain
MD	Methyl-group-donor binding domain
