# Supplementary material for: How to Unmask an Unknown: The Restriction-Modification System MhoVII of Mycoplasma hominis Expresses Two Complementary Methylation Activities in One Enzyme
Source: Int J Mol Sci. 2026 Feb 5;27(3):1591. doi: 10.3390/ijms27031591 (PMC12898733; doi:10.3390/ijms27031591)
Supplement: Supplementary file 1 [file ijms-27-01591-s001.zip › ijms-4078690-supplementary.pdf]

Supplementary Tables and Figures

Supplementary Table S1: Primers used for expression plasmid construction

PCR	primer name	primer sequence (5'-xxx-3')[a]	genomic position[b]		amplicon
			5'	3'	(bp)
rM1-A	rM1-F1_BamHI	**GGATCC**ACGTGGATTATCAAGAATAAAGATT	538598	538574	194
	rM1-R1	TTAGTTTGGTCAAATTCATAATCCCATTTC	538408	538437	
rM1-B	rM1-F2	TATGAATTTGACCAAACTAAATGGATTAA	538427	538399	168
	rM1-R2	AGGGTTATTTTTTACCCATCTTAATA	538260	538285	
rM1-C	rM1-F3	AAGATGGGTAAAAAACAACCCTATG	538281	538257	100
	rM1-R3	CCATTTTTTGTTTCCTTTTACTGCCCAAAT	538182	538211	
rM1-D	rM1-F4	AAAAGGAAACAAAAAATGGACATT	538200	538177	169
	rM1-R4	CCAATCATTCTTATTTGTATGAATAT	538032	538057	
rM1-E	rM1-F5	ACAAATAAGAATGATTGGATTTTTGAC	538049	538023	157
	rM1-R5_HinDIII	**AAGCTT**CTAGCTCATACCTTTCTATT	537899	537918	
rM1_A-E_2	rM1-F1_BamHI	**GGATCC**ACGTGGATTATCAAGAATAAAGATT	538598	538574	711
	rM1-R6_BamHI	**GGATCC**TTCTAGCTCATACCTTTCTATT	537897	537918	
rM2-A	rM2-F1_BamHI	**GGATCC**AAAATGAATAGATCACCA	537890	537873	664
	rM2-R1	CTTCTAATGTCCAGGCCTGATTAT	537231	537254	
rM2-B	rM2-F2	ATAATCAGGCCTGGACATTAGAAG	537254	537228	100
	rM2-R2	TGAAATAAATTTGATAATCCCCATTTTA	537155	537182	
rM2-C	rM2-F3	AAAAAACATAAAATGGGGATTATCAAAT	537190	537163	196
	rM2-R3_HinDIII	**AAGCTT**CCCTGCTTTTTCATAAT	537001	537017	

[a] *Bam*HI / *HinD*III restriction sites and mutated **G** in TG**A** to TG**G** codons are depicted in bold, overlapping regions used for SOE are underlined

[b] with respect to genome sequence of *M. hominis* strain SS10 (acc.-no.: CP055146)

Supplementary Table S2: Regulatory elements in the *Mho*VII gene cassette

ID / *gene*	strand	score[1]	sequence (5‘-3‘)	start[2]	end[2]
pyk	+			533826	535247
T1	+	21	CAAAACGAAAAATTAGGCTTTAACTAGGCCTATTTTTTTGTCTTGGCTTAACCG	535264	535317
T2	-	21	CAAGACAAAAAAATAGGCCTAGTTAAAGCCTAATTTTTCGTTTTGAAATTAAT	535256	535309
pal1[3]				535271	535301
T3	+	18	AAATAAAAACATGCTTTTTATTTGTTTTGACT	535354	535385
T4	-	18	AAATAAAAAGCATGTTTTTATTTTTAAAAATA	535346	535376
*R.mho*VII	-			535440	537014
*M12.mho*VII	-			536995	538601
Pro1	-	1	GATTTTTCTTCAACTTTAAATAGAAAAAATATTGATTTTTCTTCATTGGATTTTCAAGAATTTA	537333	537396
T5	-	12	ACAAGG AAACAATG CCTTGT TCGATTTTT	537390	537417
T6	+	12	TAAATT GATG AATTTA CTCTTTTCG	537578	537602
TA repeat	-		$(TA)_x$	537641	537661
Pro2	-	0.93	**TATAACTAAAATTATAAAAAATTTAATTTTGT**ATAGTCGTTATTTTCGTTTTTTTTGTTATGAT	538608	538671
T7	-	21	AAAATTTT**TATAACTAAAATTATAAAAAATTTAATTTTGT**	538640	538679
T8	+	21	AAATTTTTTATAATTTTAGTTATAAAAATTTTCTATATAAA	538648	538688
Pro3	-	0.89	AAGTAAGTGTATAAAAAAAGAGAATTAAGAAAATTTAGAGTTGAATATCTATTTGTTCCTATAT	538700	538763
pal2				538775	538810
*ser*S	-			538827	540092

[1]scores of terminator regions (T_x): strong ≥ 18–25; moderate = (12–17) and of promoter region (P_x): strong ≥ 0.9; moderate = (0.9-0.7);
[2]relative to SS10 (acc.no. CP055146.1);
[3]pal = palindrome
reverse complementary sequences underlined; **identical sequences in bold**

Supplementary Table S3: Calculation of transcript quantities of overlapping regions

	PCR	I[1]	II[2]	III[2]	IV[1]	V[2]	V[1]	VI[2]
		**Mean CT values**						
replicate 1	FBG	26.1	23.5	24.0	24.3	24.2	24.5	25.6
	SS25	29.1	26.4	25.9	27.1	26.4	26.9	-
	VO31120	31.7	26.1	26.4	26.9	26.5	27.3	-
	SS10	28.5	25.3	23.8	25.4	25.4	26.0	-
	21127	30.6	26.3	26.2	27.1	26.4	27.4	-
	10428	27.5	24.5	23.6	24.1	23.4	23.9	26.1
	1014VA	32.2	27.2	27.9	28.8	28.3	29.0	-
	SP3615	27.2	23.0	21.9	22.2	21.6	22.1	-
	1134J	29.0	25.8	25.1	26.1	25.9	26.6	-
	15562	31.6	28.6	28.0	28.7	28.2	26.6	-
	1627	30.8	27.1	27.4	28.0	27.7	28.5	-
	18281	30.2	27.3	26.5	27.4	26.3	27.1	-
replicate 2	FBG	30.5	25.3	26.8	24.3	28.0	25.1	-
	SS25	29.8	31.4	29.5	27.1	29.7	25.3	-
	VO31120	25.6	28.6	27.8	26.9	29.0	24.0	-
	SS10	29.3	31.3	30.1	25.4	31.0	27.2	-
	21127	-	32.1	32.5	27.1	32.5	29.0	-
	10428	26.5	24.3	23.8	24.1	24.5	22.3	27.1
	1014VA	-	27.6	27.2	28.8	27.1	25.4	-
	SP3615	28.3	26.3	25.3	22.2	25.3	21.3	-
	1134J	26.2	24.2	23.7	26.1	24.0	23.0	-
	15562	-	29.8	28.2	28.7	28.6	28.2	-
	1627	27.0	25.8	24.7	28.0	25.7	24.7	-
	18281	-	27.6	25.7	27.4	27.0	25.1	-

[1] Annealing temperature = 64°C

[2] Annealing temperature = 54°C

	x-fold to V					
	I	II	III	IV	V	VI
FBG	0.340	1.580	1.117	1.125	1.000	0.375
SS25	0.221	0.973	1.404	0.856	1.000	0.000
VO31120	0.049	1.301	1.083	1.333	1.000	0.000
SS10	0.186	1.091	3.010	1.521	1.000	0.000
21127	0.108	1.042	1.106	1.244	1.000	0.000
10428	0.081	0.463	0.871	0.911	1.000	0.149
1014VA	0.108	2.196	1.320	1.091	1.000	0.000
SP3615	0.029	0.388	0.807	0.936	1.000	0.000
1134J	0.190	1.110	1.759	1.495	1.000	0.000
15562	0.032	0.763	1.165	0.247	1.000	0.000
1627	0.201	1.569	1.288	1.371	1.000	0.000
18281	0.117	0.495	0.865	0.812	1.000	0.000
FBG	0.024	6.431	2.297	1.647	1.000	0.000
SS25	0.046	0.309	1.141	0.294	1.000	0.000
VO31120	0.331	1.343	2.321	0.135	1.000	0.000
SS10	0.236	0.815	1.886	3.364	1.000	0.000
21127	0.000	1.297	0.993	3.681	1.000	0.000
10428	0.056	1.149	1.647	0.296	1.000	0.167
1014VA	0.000	0.730	0.976	0.092	1.000	0.000
SP3615	0.008	0.495	1.017	0.529	1.000	0.000
1134J	0.111	0.892	1.202	0.119	1.000	0.000
15562	0.000	0.419	1.310	0.740	1.000	0.000
1627	0.198	0.880	1.905	0.100	1.000	0.000
18281	0.000	0.635	2.471	0.213	1.000	0.000
**mean**	**0.111**	**1.182**	**1.457**	**1.006**	**1.000**	**0.029**
**SA**	**0.102**	**1.180**	**0.572**	**0.907**	**0.000**	**0.084**

Supplementary Table S4: REBASE based MTases homologs and methylation frequency of corresponding motifs

Sample	REBASE Enzyme	Motif	Type	Meth.	Strand	Key	e-value	# sites	MF[1]	Prokka annotation	start	end
**4518VA_1**	M.PspGI	CCWGG	II	4mC	+	C"C"WGG(+)	1.09E-97	253	82.9	HBEAMMEN_00558	316629	317873
					-	C"C"WGG(-)		253	83.6			
	M.TacI	TCANNNNNNNRTGT	I	6mA	+	ACAYNNNNNNNTG"A"(+)	4.66E-64	111	1.4	HBEAMMEN_00708	406648	407475
					-	ACAYNNNNNNNTG"A"(-)		91	3.2			
	M.Cau700975II	GATC	II	6mA	+	G"A"TC(+)	2.69E-59	1197	74.7	HBEAMMEN_01062	624731	626017
					-	G"A"TC(-)		1197	74.0			
**4518VA_2**	M.PspGI	CCWGG	II	4mC	+	C"C"WGG(+)	1.09E-97	251	83.3	HBEAMMEN_00558	316629	317873
					-	C"C"WGG(-)		251	84.5			
	M.TacI	TCANNNNNNNRTGT	I	6mA	+	ACAYNNNNNNNTG"A"(+)	4.66E-64	110	1.4	HBEAMMEN_00708	406648	407475
					-	ACAYNNNNNNNTG"A"(-)		91	3.2			
	M.Cau700975II	GATC	II	6mA	+	G"A"TC(+)	2.69E-59	1204	74.0	HBEAMMEN_01062	624731	626017
					-	G"A"TC(-)		1204	73.4			
**1014VA_1**	M1.Hin4II	CCTTC	II	5mC	+	C"C"TTC(+)	2.64E-131	451	76.9	LBAAKCKB_00078	46978	47958
					-	C"C"TTC(-)		484	76.4			
	M.HpyAV	CCTTC	II	6mA	+	GA"A"GG(+)	1.41E-144	484	81.1	LBAAKCKB_00079	47955	49466
					-	GA"A"GG(-)		451	80.0			
	M1.NmeBI	GACGC	II	5mC	+	GA"C"GC(+)	5.72E-120	79	96.4	LBAAKCKB_00741	419082	420149
					-	GA"C"GC(-)		68	96.6			
	M2.McaCI	CCATC	II	6mA	+	CC"A"TC(+)	4.32E-41	332	84.6	LBAAKCKB_01003	577302	578075
					-	CC"A"TC(-)		371	85.6			
					+	G"A"TGG(+)		371	71.0			
					-	G"A"TGG(-)		332	67.9			
	M.TacI	TCANNNNNNNRTGT	I	6mA	+	TC"A"NNNNNNNRTGT(+)	4.66E-64	105	8.8	LBAAKCKB_01341	761986	762813
					-	TC"A"NNNNNNNRTGT(-)		107	10.1			
**1014VA_2**	M1.Hin4II	CCTTC	II	5mC	+	C"C"TTC(+)	2.64E-131	451	77.6	LBAAKCKB_00078	46978	47958
					-	C"C"TTC(-)		484	76.9			
	M.HpyAV	CCTTC	II	6mA	+	GA"A"GG(+)	1.41E-144	484	79.7	LBAAKCKB_00079	47955	49466
					-	GA"A"GG(-)		451	79.1			
	M1.NmeBI	GACGC	II	5mC	+	GA"C"GC(+)	5.72E-120	79	91.0	LBAAKCKB_00741	419082	420149
					-	GA"C"GC(-)		68	91.9			
	M2.McaCI	CCATC	II	6mA	+	CC"A"TC(+)	4.32E-41	332	34.1	LBAAKCKB_01003	577302	578075
					-	CC"A"TC(-)		371	34.1			
						G"A"TGG(+)		371	30.5			
						G"A"TGG(-)		332	29.6			
	M.TacI	TCANNNNNNNRTGT	I	6mA	+	TC"A"NNNNNNNRTGT(+)	4.66E-64	105	4.3	LBAAKCKB_01341	761986	762813
					-	TC"A"NNNNNNNRTGT(-)		107	4.8			

[1]Methylation frequency

Supplementary Table S5: Motif abundance in selected *M. hominis* isolates

	***M. hominis* isolate**	**number of motifs/genome**		
		**GATC**	**GATG**	**CATC**
**RM.*Mho*VII +**	FBG	574	163	163
	SS25	579	160	160
	VO31120	573	163	163
	SS10	589	162	162
	21127	567	163	163
	10428U	599	165	165
	1627	586	162	162
	1014VA	568	166	166
	SP3615	595	162	162
	1134J	562	160	160
	15562	569	161	161
	**mean**	**578**	**162**	**162**
**RM.MhoVII -**	4518VA	582	161	161
	8958VA	649	160	160
	A136	576	163	163
	PG21	577	161	161
	2740	630	162	162
	7316U	578	160	160
	16753VA	601	160	160
	**mean**	**599**	**161**	**161**

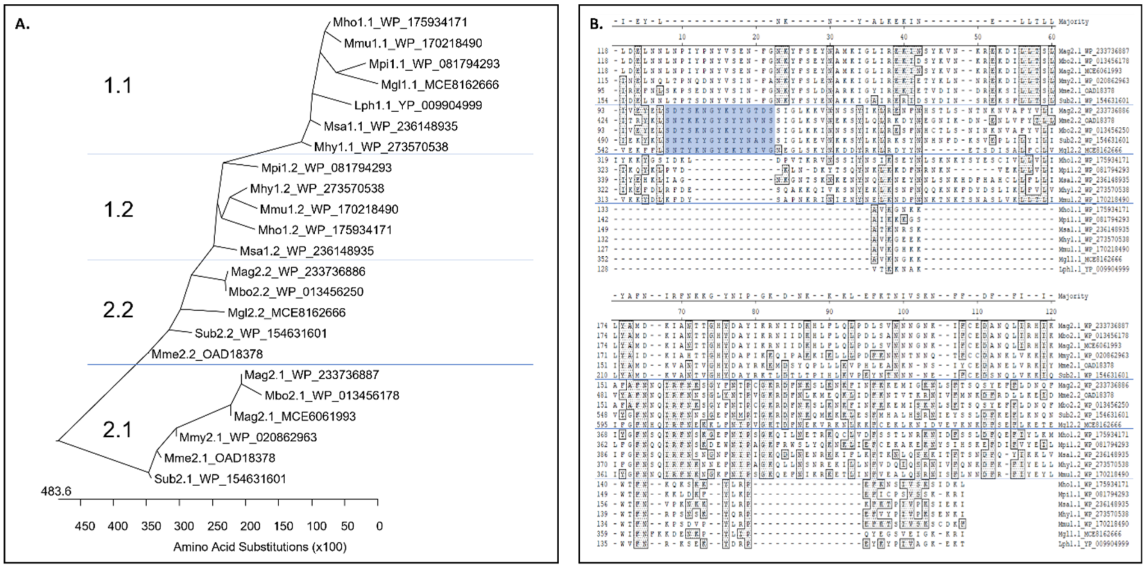

Supplementary Fig. S1: Phylogenetic tree (A) based on a multiple sequence alignment (B) of the TRD region of *Mho*VII MTase homologs.

(A) The phylogenetic tree was constructed with MegAlign 5.08; Labelling consists of abbreviation of the organism, numbering of MTase region and accession numbers of the protein (see Table 2). Branches of *Mho*VII MTase TRD regions are numbered according to Fig. 2/Table2. (B) In multiple sequence alignment the (2.2)-specific region is coloured in blue.

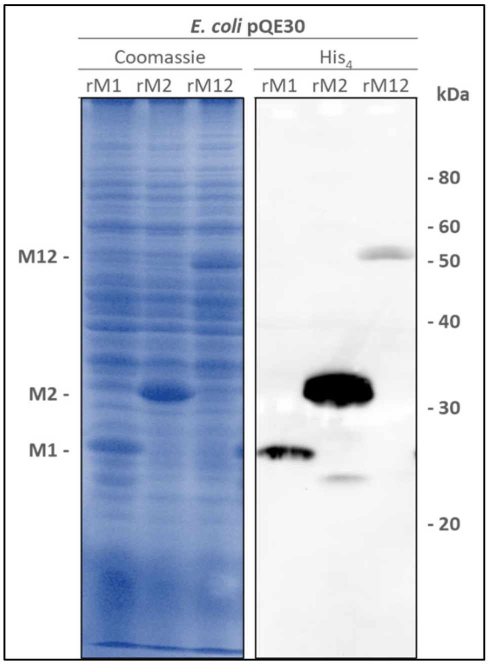

Supplementary Fig. S2: Expression profiles of *E. coli* clones

Lysates of *E. coli* DH5aF'IQ-clones (corresponding 0.5 mL cultures/lane) were subjected to 12% SDS-PAGE; expressing rM1, rM2 or the rM12-fusion protein. Protein patterns were either stained with Coomassie Brilliant Blue (left) or anti His_4-immunostained (right). Molecular weight of the proteins: rM1=29.2 kDa, rM2=36.5 kDa rM12 = 65.7 kDa

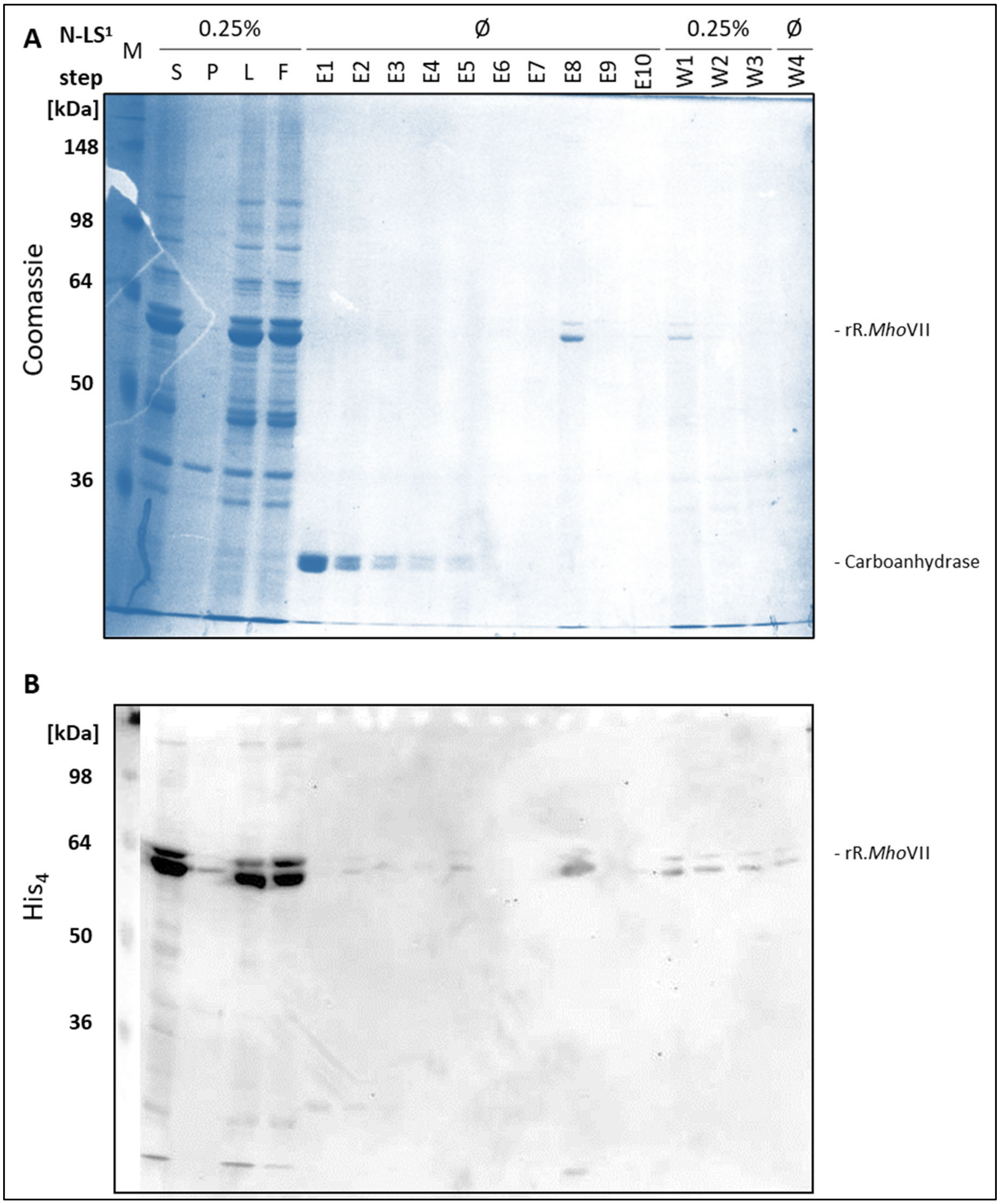

Supplementary Fig. S3: Coomassie and His_4-staining of rR.*Mho*VII purification

rR.*Mho*VII was purified according to the chapter "Cloning and heterologous expression of *Mho*VII proteins". The labelling of the lanes is according to the purification steps: Marker (M), starting material (S), pellet (P), lysate (L), flow-through (F), washings (W1-W4) and elution (E1-E10). [1]n-lauryl sarcosine. Molecular weight of rR.*Mho*VII: 63.3 kDa.

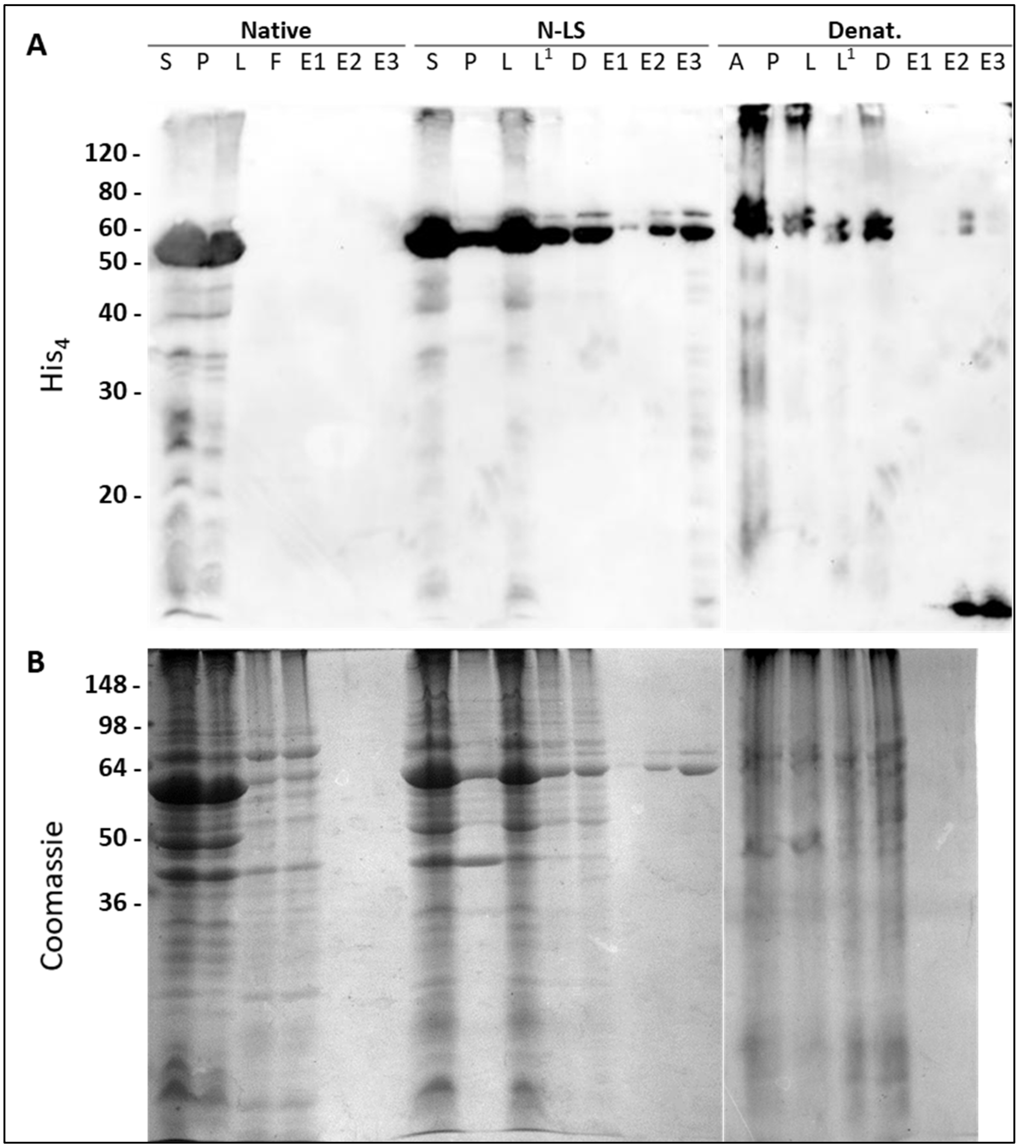

Supplementary Fig. S4: Solubility Assays of rR.*Mho*VII

E. coli disrupted under native conditions, supplemented with n-lauryl sarcosine (NLS) or under denaturing conditions (Denat.; 6 M guanidinium chloride and 8 M urea) were separated on 12% SDS gels and stained with His_4-antibodies (top) or Coomassie-staining (bottom). The labelling of the lanes is according to the purification steps: Marker (M), starting material (S), pellet (P), lysate (L), flow-through (F), washings (W1-W4) and elution (E1-E10). [1] = 1:10 dilution of the lysate. Molecular weight of rR.*Mho*VII: 63.3 kDa.

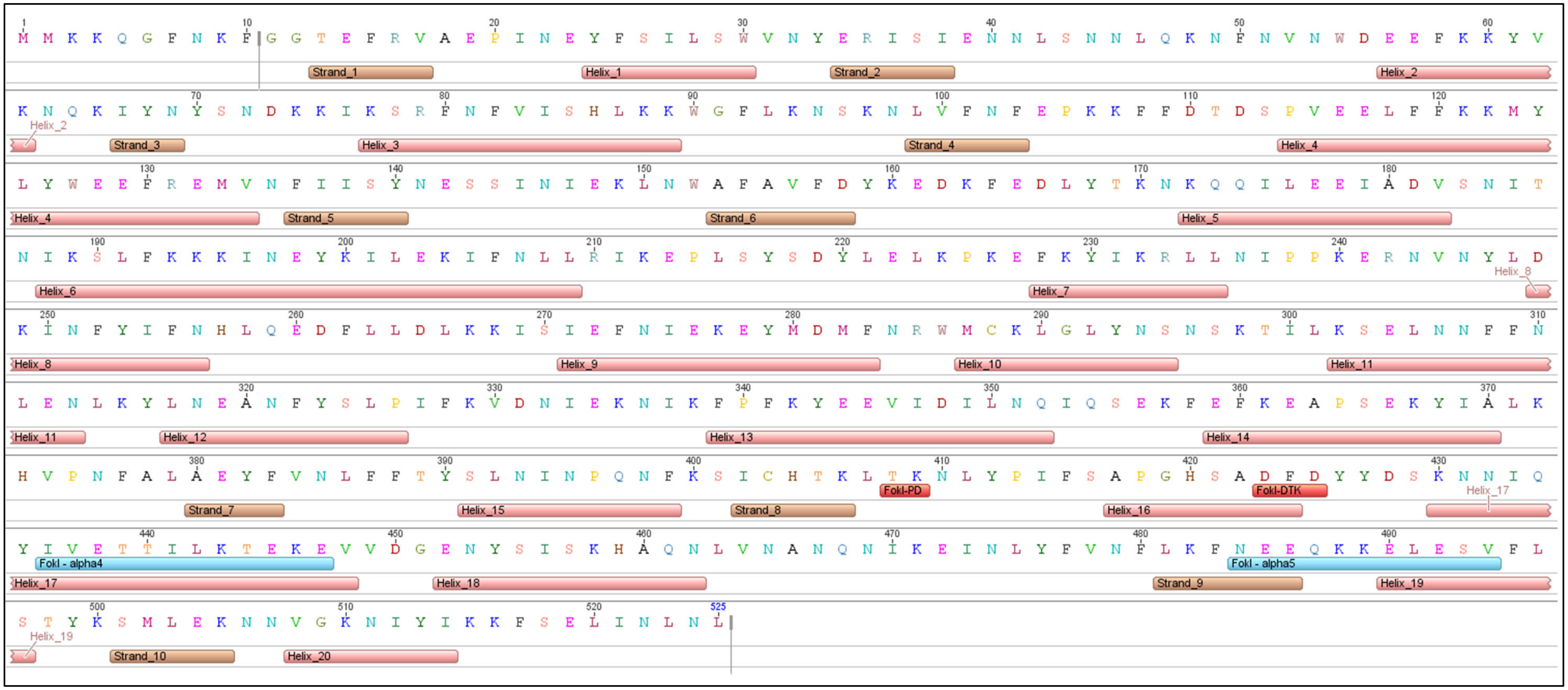

Supplementary Fig. S5: Secondary structure of R.*Mho*VII

Amino acid sequence of R.*Mho*VII. Secondary structures predicted with Phyre2.2 are annotated as α-helices (rose) or β-sheets (brown). The corresponding length and position of the α4 and α5 helix of *Fok*I is marked in blue and the active site of *Fok*I in red.

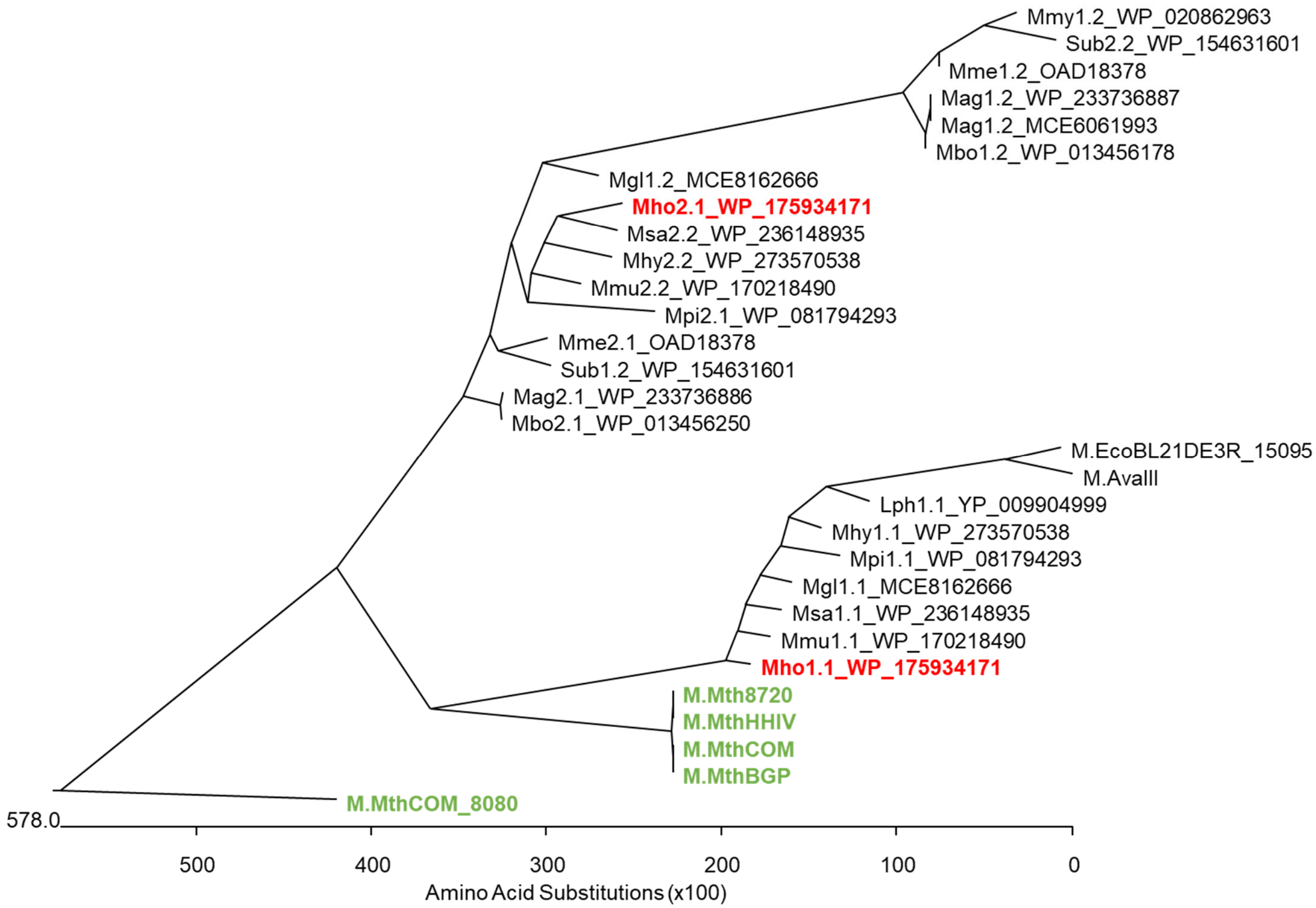

Supplementary Fig. S6: Phylogentic tree of RM.*Mho*VII homologs and *M. thermoacetica* MTases.

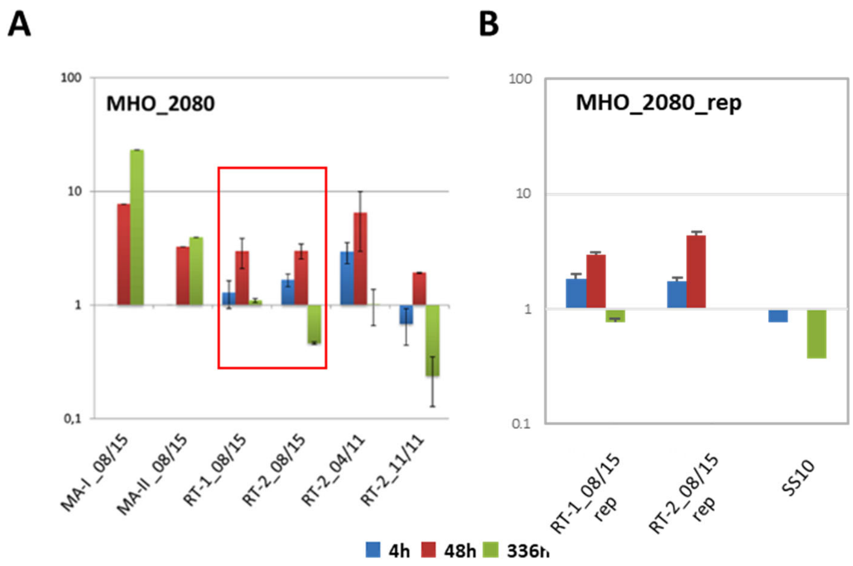

Supplementary Fig. S7: Transcript levels of MHO-2080 in different stages of HeLa cell infections.

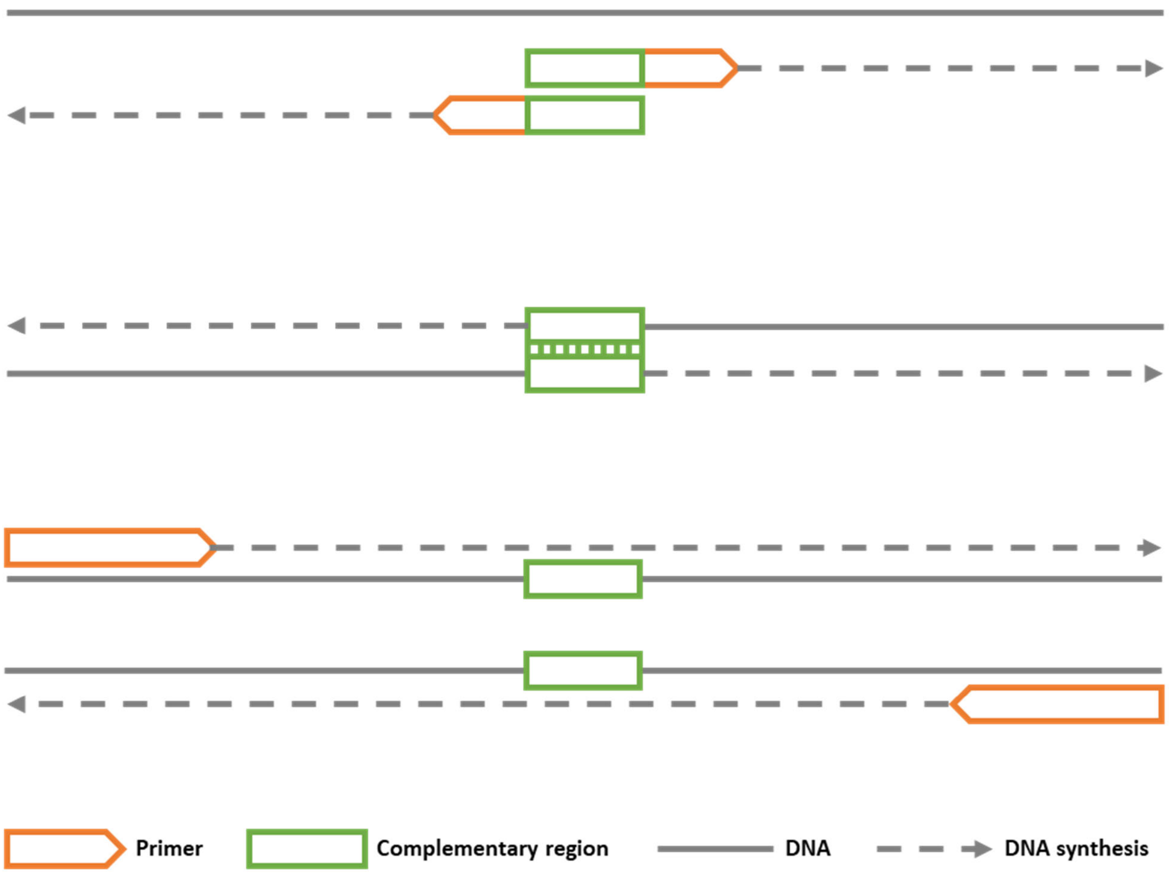

1. **PCR reaction with interal primers**
   - These primes contain a complementary region (green)
2. **Overlap extension of PCR fragments without primers**
   - Purified PCR products are mixed without added primers
   - Annealing temperature = T_m of the overlapping complementary region
   - The overlapping regions anneal, and DNA polymerase extends each fragment using the opposing fragment as template, generating a full-length product
3. **PCR with external primers**
   - External primers are used to amplify the full-length gene
4. **Repeat steps according to the number of fragments**

Supplementary Fig. S8: Principle of SOE method.

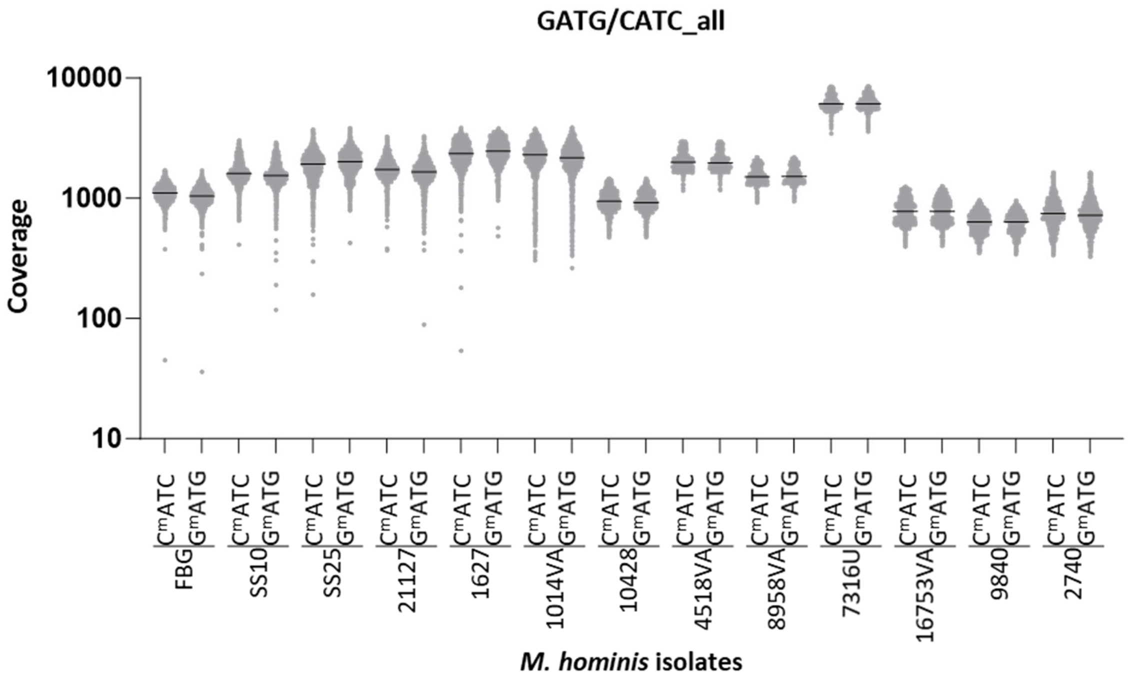

Supplementary Fig. S9: Coverage of 6mA methylated adenines of G^{m}ATG and C^{m}ATC motifs in *M. hominis*.

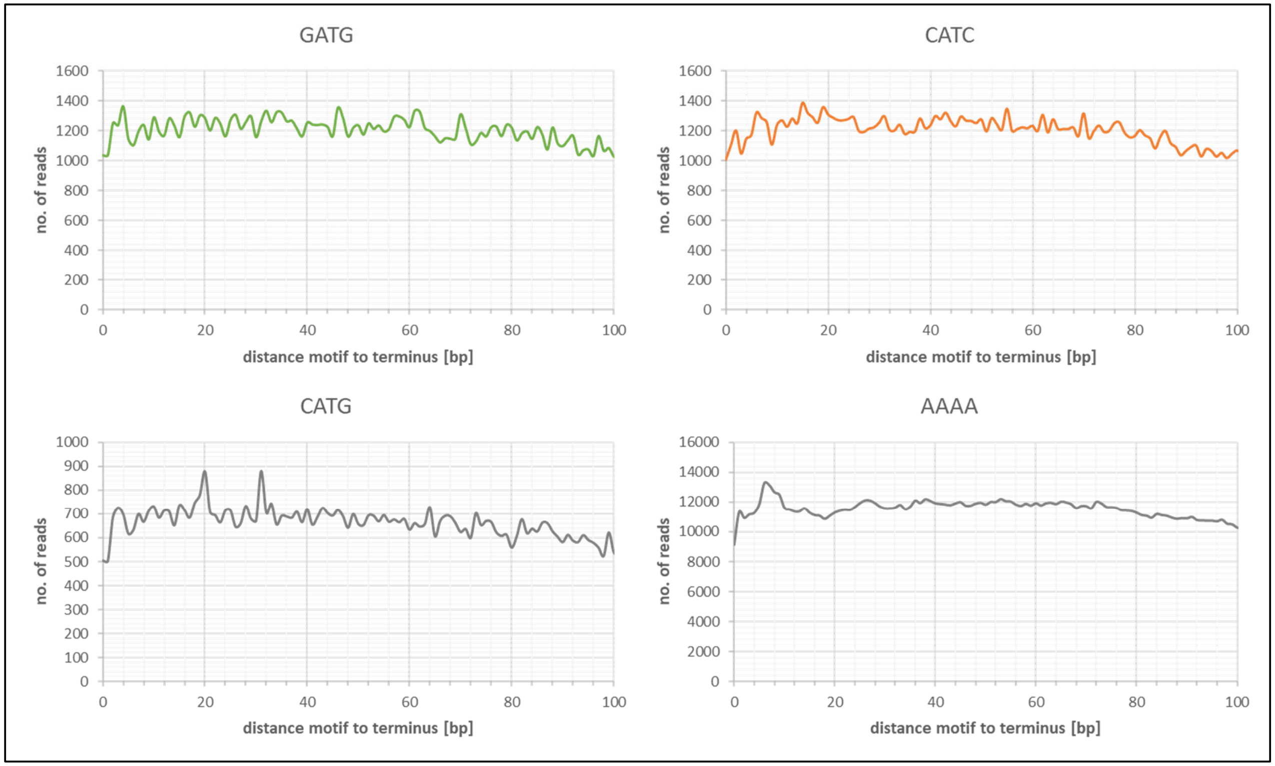

Supplementary Fig. S10. Plot of 100 bp 3'-ends of nanopore reads from R.*Mho*VII restricted DNA. For each Oxford Nanopore read containing at least one complete GATG, CATC, CATG or AAAA motif, the distance between this motif and the read terminus was calculated. The number of reads is plotted as a function of the distance from the read end (0–100 bp) to the respective motif.
